# COVID-19 transmission dynamics underlying epidemic waves in Kenya

**DOI:** 10.1126/science.abk0414

**Published:** 2021-10-07

**Authors:** Samuel P. C. Brand, John Ojal, Rabia Aziza, Vincent Were, Emelda A. Okiro, Ivy K. Kombe, Caroline Mburu, Morris Ogero, Ambrose Agweyu, George M. Warimwe, James Nyagwange, Henry Karanja, John N. Gitonga, Daisy Mugo, Sophie Uyoga, Ifedayo M. O. Adetifa, J. Anthony G. Scott, Edward Otieno, Nickson Murunga, Mark Otiende, Lynette I. Ochola-Oyier, Charles N. Agoti, George Githinji, Kadondi Kasera, Patrick Amoth, Mercy Mwangangi, Rashid Aman, Wangari Ng’ang’a, Benjamin Tsofa, Philip Bejon, Matt. J. Keeling, D. James. Nokes, Edwine Barasa

**Affiliations:** 1 Kenya Medical Research Institute (KEMRI)-Wellcome Trust Research Programme (KWTRP), Kilifi, Kenya; 2 The Zeeman Institute for Systems Biology and Infectious Disease Epidemiology Research (SBIDER), University of Warwick, Warwick, UK; 3 School of Life Sciences, University of Warwick, Warwick, UK; 4 London School of Hygiene and Tropical Medicine (LSHTM), London, UK; 5 Health Economics Research Unit, KEMRI-Wellcome Trust Research Programme, Nairobi, Kenya; 6 Population Health Unit, Kenya Medical Research Institute-Wellcome Trust Research Programme, Nairobi, Kenya; 7 Centre for Tropical Medicine and Global Health, Nuffield Department of Medicine, University of Oxford, Oxford, UK; 8 Department of Infectious Diseases Epidemiology, London School of Hygiene and Tropical Medicine, London, UK; 9 Ministry of Health, Government of Kenya, Nairobi, Kenya; 10 Presidential Policy and Strategy Unit, The Presidency, Government of Kenya; 11 Mathematics Institute, University of Warwick, Warwick, UK

## Abstract

Policy decisions on COVID-19 interventions should be informed by a local, regional and national understanding of severe acute respiratory syndrome coronavirus 2 (SARS-CoV-2) transmission. Epidemic waves may result when restrictions are lifted or poorly adhered to, variants with new phenotypic properties successfully invade, or infection spreads to susceptible subpopulations. Three COVID-19 epidemic waves have been observed in Kenya. Using a mechanistic mathematical model, we explain the first two distinct waves by differences in contact rates in high and low social-economic groups, and the third wave by the introduction of higher-transmissibility variants. Reopening schools led to a minor increase in transmission between the second and third waves. Socioeconomic and urban-rural population structure are critical determinants of viral transmission in Kenya.

After the first polymerase chain reaction (PCR)-confirmed case of COVID-19 in Kenya on 13 March 2020, the Kenyan government rapidly introduced measures aimed at suppressing severe acute respiratory syndrome coronavirus 2 (SARS-CoV-2) transmission in the country. These measures included the closure of international borders, with the exception of cargo movement; closing of schools and other learning institutions; a ban on social gatherings and meetings; closure of places of worship, bars, and restaurants; a dawn-to-dusk curfew; mandatory wearing of masks in public places; physical distancing guidelines, including on public transportation; and restrictions on movement into or out of counties with high infection rates, including the two main Kenyan cities, Nairobi and Mombasa (1) (Fig. 1). Despite these measures, the rate of new COVID-19 cases grew in Kenya, indicating that measures had not been enough to consistently push the effective reproduction number *R*(*t*) below 1. Moreover, serological surveillance indicated that a higher-than-expected fraction of the Kenyan population had been exposed to SARS-CoV-2 given the case reports at the time: June 2020 adjusted seroprevalences, based on blood donor samples from the Kenya National Blood Transfusion Services (KNBTS), were 5.6% for Kenya, 8% for Mombasa, and 7.3% for Nairobi (2).

Detected COVID-19 incidence in Kenya first peaked in early August 2020 during a period of relaxation of measures: the end of the Nairobi and Coastal counties (including Mombasa) lockdown (7 June 2020), and the resumption of international air travel (1 August 2020). A single-wave epidemic in Kenya peaking within 100 to 200 days after SARS-CoV-2 introduction into the country was initially predicted, based on assumptions that included a single population group and the development of immunity to reinfection (3–6). However, second and third waves occurred in mid-November 2020 and in March 2021, respectively. Multiple waves of COVID-19 incidence in high-income country settings have usually been associated with a relaxation of previous restrictions—for example, in the United Kingdom (7). More recently, the emergence of new variants has been associated with further waves of infection (8). In Kenya, and other countries in Africa, a temporal association between relaxation of restrictions and subsequent waves is implausible. Understanding the causation of such multiple waves is critical for forecasting hospitalization demand and the likely effectiveness of interventions, including vaccination strategy.

There are multiple potential explanations for sequential wave dynamics in COVID-19 incidence, which might be acting singly or in concert: social clustering (9), changing adherence to measures over time (7), seasonal effects on transmission (10), reopening of places of learning (11), lower transmission rates in rural settings leading to later peaks in those areas (4), and waning immunity after an infection episode, as well as the introduction of new SARS-CoV-2 variants that are more transmissible than previous strains and/or evade prior immunity acquired by natural infection (12). The decrease in cases following the peak of the first wave occurred at a time of relaxation of social distancing measures in Kenya (Fig. 1). Hence, the end of the first wave cannot be explained by the imposition of nonpharmaceutical interventions. In this work, we present evidence that the most plausible explanation for the pattern of cases and seroprevalence observed in Kenya is a combination of differential adherence to measures between sub-populations that we identify with lower and higher socioeconomic status (SES) in 2020 followed by a sharp increase in virus transmissibility in 2021, consistent with that observed in other countries affected by variants of concern, e.g., the United Kingdom (13) and India (14). Previous studies undertaken in sub-Saharan Africa at the level of individual country (4) or pan-African exploring the impact of climate (15) have not had the opportunity to integrate longitudinal PCR, serology, and Google mobility data.

We developed a county-specific, two-SES group, SEIRS-type transmission model, using a waning immunity rate derived from recent studies on the protectiveness of a natural infection to future reinfection (16–19). Our model includes, for each Kenyan county, (i) a SEIRS transmission model predicting new infections on each day, socioeconomic group, and county, which accounts for assortativity in infections— that is, the propensity for infected individuals to cause more intragroup infections compared to intergroup infections; and (ii) an observation model reflecting the data streams: PCR testing (positive and negative results), seroprevalence surveys, Google mobility data, and determined COVID-19 deaths. The model developed for this study differs from the standard SEIRS model with homogeneous mixing, adding the impact of new variants as detected by genomic surveillance and allowing the model to fit two socioeconomic groups in counties where this was supported by the data streams. We used a hierarchical approach to inferring the underlying epidemic trajectories in each of the 47 Kenyan semiautonomous counties by the following three steps: (a) grouping counties by similarity over a range of sociological and epidemiological metrics using machine learning; (b) for the 11 counties with a relatively high density of serology tests, we jointly inferred epidemiological model parameters for each county, e.g., (i) baseline *R*_0_ for the county, (ii) the effect of schools being open on *R*(*t*), (iii) the increase in transmissibility in February 2021 when B.1.1.7 lineage (Alpha variant) SARS-CoV-2 was first detected in Kenya (20), (iv) the fraction of the population in the higher SES group in each county and their assortative mixing rate, and (v) the fraction of cases reported for the county using Hamiltonian Markov chain Monte Carlo (21) with mildly informative priors; and (c) we inferred model parameters for the remaining 36 counties using informative priors for reporting fractions derived from a synthesis of the posterior distributions of counties grouped as similar to that county (see supplementary materials for details). We conducted formal model selection to compare one, two, and three SES group models, finding that the one-group model was an inadequate fit to the data, and the three-group model was not an improvement on the two-group model (see supplementary materials). We also conducted sensitivity analysis for different assumptions on waning immunity, finding consistent results for a range of scenarios (see supplementary materials).

The two-SES group transmission model was able to capture the timing and intensity of all three waves of Kenyan COVID case incidence and the trend of increasing proportion seropositive among Kenya National Blood Transfusion Service (KNBTS) donors (Fig. 2). We also validated the fitted model by comparing forecasts of seropositivity rates with those from data not used to infer model parameters. We used rounds 1 and 2 of the seropositivity survey using KNBTS donors for model parameter inference, collected during Mayto September 2020. Estimated seroprevalence among the Kenyan population, derived from the fitted two-SES group transmission model, was in good agreement with the out-of-sample round 3 of KNBTS seroprevalence data, collected January to March 2021 (Fig. 2). The Nairobi-specific epidemic trajectory inferred in this study agrees with seroprevalence estimates from a randomized survey from Nairobi and is congruent with the observation that it was public hospitals in Nairobi (favored by lower SES groups) that came under pressure in the first wave, whereas the second wave showed increased admission to private health facilities (figs. S7 and S8). As well as capturing the past trends of case reporting and seropositivity in Kenya, the fitted two-SES group transmission model accurately predicts the daily rate of new confirmed COVID-19 cases reported by the Kenyan Ministry of Health for the month after the censoring date of the PCR test data used to infer model parameters (Fig. 2).

The two-SES group transmission model reconciled the apparent paradox between evidence of the effectiveness of the rapidly introduced Kenyan measures in reducing mobility out of the home among Kenyan smartphone users, which was close to that observed in European and North American countries (fig. S1), and that case rates and fatality rates display two distinct waves in Kenya in 2020. The model provides an explanation for the end of the first wave through the depletion of susceptibles in geographically distinct, largely urban, subpopulations of lower SES. In some Kenyan counties (e.g., the urban counties Nairobi and Mombasa, and some of the semi-urban counties), we infer that a substantial group of people belong to the higher SES group whose mobility is well represented by Google smartphone data; a combination of school closures and reduction in mobility (by 44.5%; see supplementary materials) reduced the effective reproduction number sufficiently that newly infected people among the higher SES group were on average generating less than one secondary infection by April 2020 (Fig. 3). In the counties where the model finds evidence for distinct two-group dynamics (fig. S13), the model predicts low rates of intergroup infection transmission (posterior mean for the assortativity parameter estimates of 2 to 40 disassortative infections per 1000 potential infection events). We believe this can be ascribed to pandemic-induced changes in social behavior that enhanced intra-SES group infectious contacts (such as longer contact durations in families or local communities) and decreased inter-SES group infectious contacts as a result of, for example, avoiding public transport and cancelling domestic staff visits. The growth rate in cases, and relatively high levels of seroprevalence among KNBTS donors, are explained by the rest of the population in the lower SES group having *R*(*t*) > 1 into May and June 2020 (Fig. 3). The model inferred that the reduction in mobility among the lower SES group was substantially less than among the higher SES group: The posterior mean estimate for reduction in mobility among the lower SES group in Nairobi was 13.8% [confidence interval (CI) 11.3–17.5%)] and in Kenya’s second city Mombasa was 18.9% (CI 17.4–20.4%); posterior mean estimates for lower SES group mobility reduction across all 47 Kenyan counties had a median of 15.7% [interquartile range (IQR) 10.9–19.6%]. We assumed that school closures reduced *R*(*t*) for both SES groups equally. The inferred reduction in *R*(*t*) due to schools closing varied from county to county, and the median reduction in *R*(*t*) over counties was 13.5% (IQR 4.3–23.7%); the Nairobi estimate for school closure effect was 23.8% (CI 16.5–31.6%), and the Mombasa estimate for school closure effect was 20.2% (CI 15.2-25.2%) (Fig. 3).

The second wave in Kenya in 2020 was due to the superimposition of two trends: (i) in mainly urban areas, a second wave was triggered by the higher SES group returning to pre-COVID-19 mobility patterns by early November 2020 (fig. S1) and, therefore, *R*(*t*) was >1 for the higher SES group (Fig. 3, top and bottom left); and (ii) in more rural areas, the inferred size of the higher SES group was small, and *R*(*t*) was low but persistently >1 for the lower SES group [*R*(*t*) ~ 1 to 1.5] until November 2020 (Fig. 3, bottom right, and fig. S13; see supplementary materials). Low rates of mobility somewhat shielded the higher SES group from infection in the first wave among the lower SES group. Therefore, the lower SES group, in cities, suffered two waves in 2020, whereas the higher SES group effectively suffered one wave that peaked in late 2020 (Fig. 4). The overall detection rate was determined in part by the number of PCR tests performed each day, and this rate dropped in September 2020 (fig. S4). A consequence of the drop in the testing rate was that the case reporting shows a much sharper delineation between the first two waves (Fig. 2) than the underlying inferred infection rates (Fig. 4), which reveal only a moderate dip in infections in August–September 2020. By accounting for the delay between infection and COVID-19 fatality, and fitting SES group-specific infection-fatality-detection ratios (IFR detection; see materials and methods and supplementary materials) to each county, we found reasonable agreement between the predicted and observed timings of peak fatality rates in Kenya (Fig. 4). Overall, our model-based estimate was that only 11% of the total Kenyan population was in the higher SES group, whose mobility was well-described by Google mobility data, with the highest concentration of higher SES group individuals in the two main cities: 43.4% of the Nairobi population (CI 35.4–49.2%) and 40.3% of the Mombasa population (CI 35.0–45.4%). Additionally, we estimate that infections among the higher SES group were substantially more likely to be detected than among the lower SES group: The odds ratio for Nairobi for detection per case between higher and lower SES was 4.5 (C11.5–17.9), and for Mombasa for detection per case between higher and lower SES was 4.8 (CI 3.2–6.8). The odds ratio between detection per infection in the two SES groups was inferred to be even more extreme across Kenya as a whole, with substantial variation from county to county: The median odds ratio estimate over counties was 18.5 (counties estimate IQR 2.5–27.9), although most counties had a small number of people in the higher SES group.

Fully reopening schools in January 2021 was associated with a slight increase in cases and deaths in Kenya, with a peak in January and early February 2021 (Figs. 3 and 4). However, school reopening does not explain the third wave in Kenya observed in March and April 2021, which was considerably larger than the increase in January and February 2021. The two-SES group model was not a sufficient explanation for a third wave, neither was loss of immunity or any detectable trend in mobility. The first cases of the more transmissible Alpha variant B.1.1.7 were identified in Kenya from mid-January 2021 (20). We investigated if the data supported an increase in transmissibility per infected person starting at the beginning of February 2021, as well as an influx of new exposed individuals representing domination of wild-type strains of SARS-CoV-2 by a fitter new variant. In the Kenyan urban counties, we found a credible range of increase in transmissibility of 15.0 to 46.6% [Nairobi 32.5% (CI 18.1–46.6%); Mombasa 22.8% (C115.0-31.2%)], and this was reflected in increased transmissibility estimates across Kenyan counties: The median over county estimates was 46.1% (IQR 31.6–72.9%). The fitted model predicted that this large increase in transmissibility will push the overall exposure to SARS-CoV-2 in Kenya from a back-calculated estimate of 53.5% in February 2021 to 78.1% by June 2021 (Fig. 2). The rate of seroreversion—that is, the loss of detectable antibodies rather than necessarily loss of protective immunity—has been identified as an important quantity for estimating population exposure prevalence from serological data (22). Because the serological data used for parameter inference was collected within 7 months of the first identified case in Kenya, we assumed that seroreversion was negligible over this period. However, assuming no future seroreversion led to closer agreement between model back-calculation and round 3 KNBTS data than assuming a median 1 year between infection and seroreversion (Fig. 2); that is, our modeling does not support the need to incorporate seroreversion to capture the true population exposure over the time scale of a year, unlike for Buss et al. (22). This finding highlights that seroreversion rate depends on the serological assay used (23) and cannot necessarily be extrapolated between settings. A negligible seroreversion rate may be more applicable for the enzyme-linked immunosorbent assay used in Kenya, where the cut-offs prioritize specificity over sensitivity (2, 24).

Our modeling exercise provides a credible mechanistic interpretation of the three waves of COVID-19 in Kenya. We hypothesize the presence of two SES groups in each county and allow the model freedom to fit the relative proportion in each by county, inferred from locally collected PCR and serological test data. The model results support the notion of two SES groups in urban settings defined by highly assortative mixing (Nairobi, Mombasa, and predominantly counties near Nairobi), whereas for most rural counties, mixing was inferred to be less assortative and/or effectively all the population is in a single SES group (fig. S13). We invoke two key underlying assumptions. First, a stratified population differing in mobility (associated with lower and higher SES), and second, increased virus transmissibility compatible with competitive succession of a SARS-CoV-2 variant of concern in wave 3. A key simplifying assumption in this modeling study is that we assumed that the diversity of behaviors across the population in each Kenyan county can be reduced to stratifying into two groups with assortative mixing favoring transmission within group, and identifying these groups into lower and higher SES groups on the basis of similarity to mobility trends among smartphone users. We believe that this is a well-evidenced hypothesis for Kenya. Marked social and economic structuring has been described in Kenya; 36% of the population live below the national poverty line (25), and 55% live in informal settlements (26). Further, 83% of Kenya’s labor market is informal, characterized by unstable and unpredictable daily wages (27). Lower socioeconomic groups have been identified as vulnerable to SARS-CoV-2 in the Global South because of residence in informal settlements at high population density, reduced access to sanitation, and dependence on informal employment requiring daily mobility (28,29). By contrast, the higher SES group with job security can work from home, socially distance, and readily access water and sanitation, thereby decreasing transmission. In Kenya, Google mobility data from smartphone users indicate a sharp decline in movement to settings outside of the home (fig. S1). We found that the two-SES group model used in this study was able to capture pattern of cases and seroprevalence in Kenya over the first three waves, despite radically simplifying the underlying social structure.

We predict the proportion of the Kenyan population exposed to SARS-CoV-2 to be greater than 75% by the beginning of June 2021 (Fig. 2), corresponding to around 39 million people. However, fewer than 4000 confirmed COVID-19 deaths and 180,000 confirmed SARS-CoV-2 infections have been identified as of 1 June 2021. We found that people among the lower SES group were likely to be even more undersampled than people among the upper SES group, as well as identifying wide regional variation in the detection rate. These results emphasize the necessity of community surveys of SARS-CoV-2 prevalence and exposure, and an investigation into the underreporting of mortality and severe disease due to COVID-19. Multiple waves have been observed in many other African countries that do not appear to be completely explained by the timing of restrictions, and because they also have in common similar socioeconomic groupings in urban centers, we speculate that the explanations found to be plausible in our model for Kenya may apply more widely.

The high population exposure suggests that a fourth COVID-19 wave in Kenya before the end of 2021 would only be likely if (i) a variant arises with substantial further enhancement in transmissibility or immune escape, such as the B.1.617.2 Delta variant (30), or (ii) there is substantial waning of immunity in those previously exposed. We predict that ~75% of the Kenyan population has been exposed to SARS-CoV-2 by June 2021. This will mitigate the death rate that might be expected in the future, but taking together (i) the markedly increased transmissibility of Delta variant, (ii) the potential for reinfection, and (iii) the experience of other countries despite prevalent vaccination, this scenario is entirely consistent with a fourth wave in Kenya. We conclude that our analysis, which triangulates PCR test, seroprevalence, and mobility and genomic data to develop a coherent explanation of the transmission dynamics of COVID-19, provides insight of public health importance in Kenya, including targeting health care capacity and pharmaceutical and nonpharmaceutical interventions.

## Supplementary Material

Supplementary Materials

## Figures and Tables

**Fig. 1:**
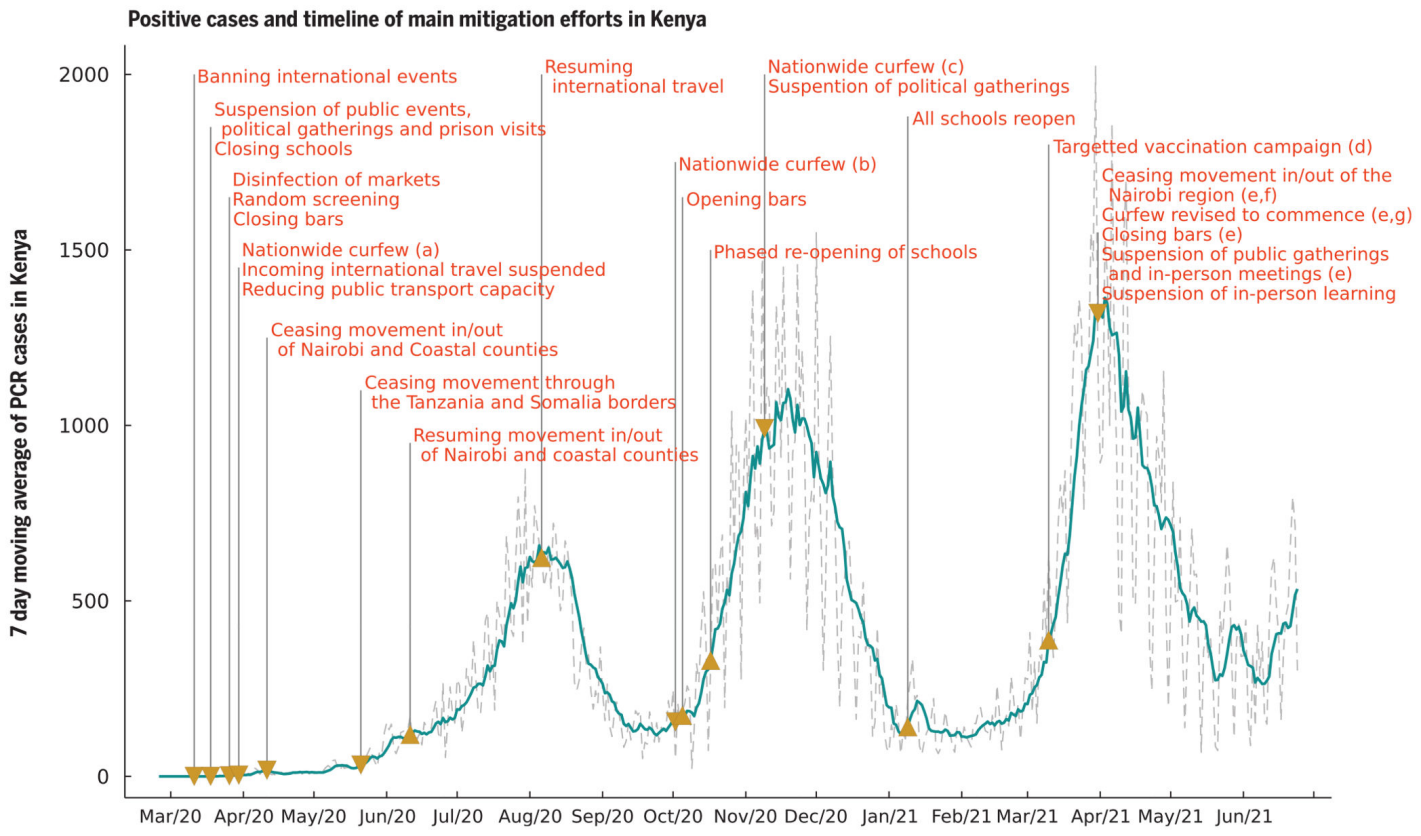
Seven-day moving average of daily positive PCR tests from the Kenyan national linelist and a timeline of the main mitigation events applied by the Kenyan government representing tightening (down-arrow) and relaxation (up-arrow) of measures. (a) Curfew from 7 p.m. to 5 a.m.; (b) curfew from 11 p.m. to 4 a.m.; (c) curfew from 10 p.m. to 4 a.m.; (d) front-line workers and individuals older than 58 years (~1.2 million doses); (e) the region includes Nairobi, Kajiado, Machakos, Kiambu, and Nakuru; (f) this restricted movement into and out of the block of counties in (e) but not between these counties; (g) curfew from 8 p.m. to 4 a.m.

**Fig. 2:**
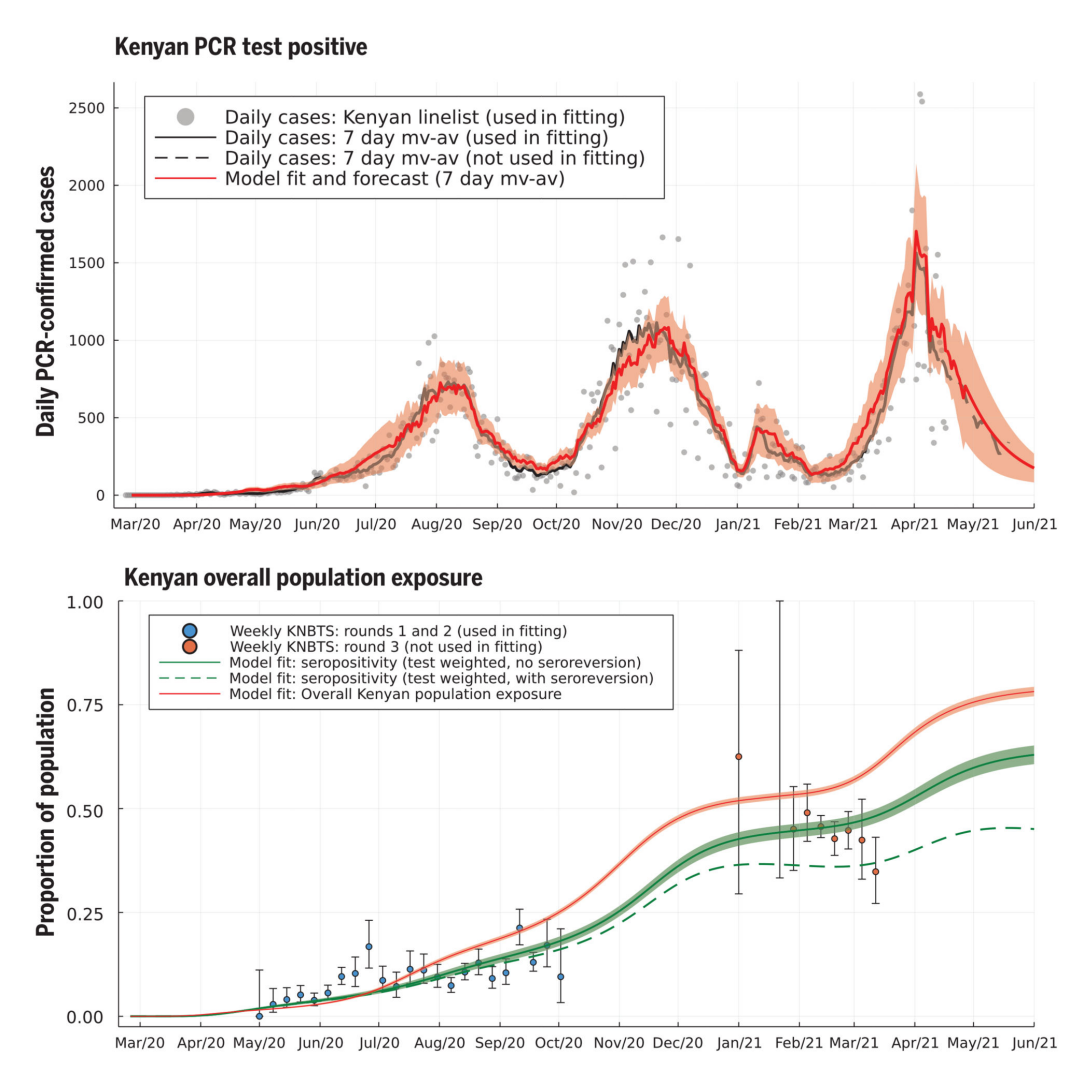
Daily PCR-confirmed COVID-19 cases (top) and weekly serology estimates from KNBTS donors with overall attack rate estimates (bottom). Shown are daily numbers of PCR test positives from the Kenyan national linelist (top; gray dots are daily reports used in fitting the model, curves are 7-day moving averages). The model prediction for the 7-day moving average of daily case incidence (top; red dashed curve, shading shows 3-s intervals) were derived from inference on the county-specific linelist PCR data and rounds 1 and 2 of the KNBTS serology survey (bottom; blue dots). Predictions before mid-April 2021 are back-calculations using known numbers of PCR tests per day, whereas after mid-April 2021, model predictions are forecasts that also estimate the number of PCR tests that will occur per day in each county. We show the next month of PCR test positive data, not used in fitting, as a validation of the model’s short-term predictive accuracy (top; black dashed curve). Back-calculated model estimates of seropositivity (bottom; green solid curve) are shown with round 3 of the KNBTS serology survey data (bottom; red dots, not used in model inference). We also show back-calculated estimates of seropositivity under the assumption that median time to seroreversion (loss of detectable antibodies rather than loss of immunity) from infection was 1 year. Model estimates of overall Kenyan seropositivity are adjusted from county-specific estimates by weighting by number of serology tests in each county (over KNBTS rounds 1 and 2). The overall estimated Kenyan attack rate (population exposure) is shown as unweighted (bottom; red curve).

**Fig. 3:**
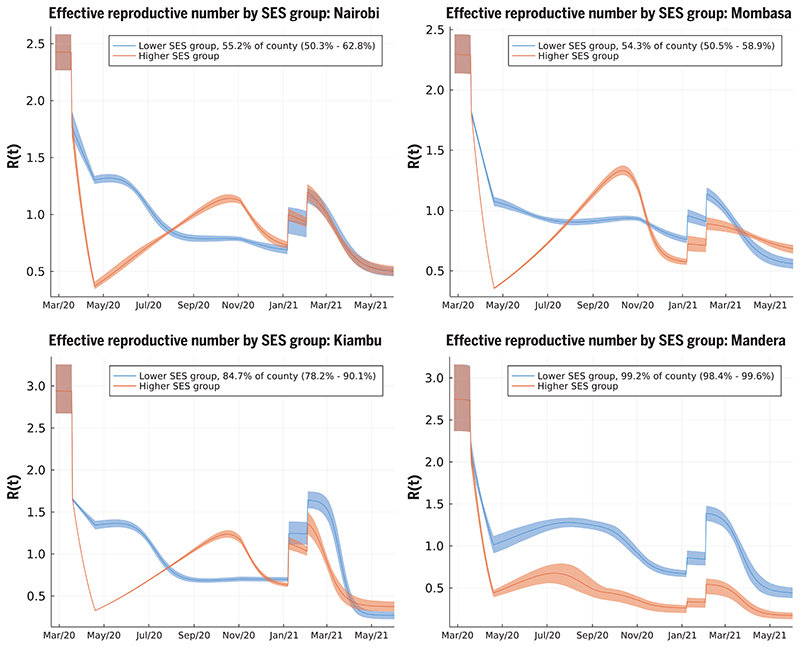
Effective reproduction number over time [*R*(*t*)] for lower and higher SES groups in four representative counties. These include Nairobi (top left), Mombasa (top right), Kiambu (bottom left), and Mandera (bottom right). Nairobi and Mombasa are Kenya’s two largest cities and form fully urban counties; Mandera county has a largely rural population and is remote from the main urban conurbations; Kiambu county borders Nairobi and has a ~60% urban population. The transmission model infers the proportion of the population in each SES group in each county. The highest proportion of higher SES group individuals are inferred to be in Nairobi and Mombasa out of all counties, whereas for Mandera county, very close to all individuals are inferred as being in the lower SES group, and the model effectively defaults to one group SEIRS transmission. The model inference for *R*(*t*) in Kiambu represents a county between these two extremes. In each county, the first discontinuous increase in *R*(*t*) is due to schools reopening, and the second is due to more transmissible variants becoming dominant in transmission.

**Fig. 4:**
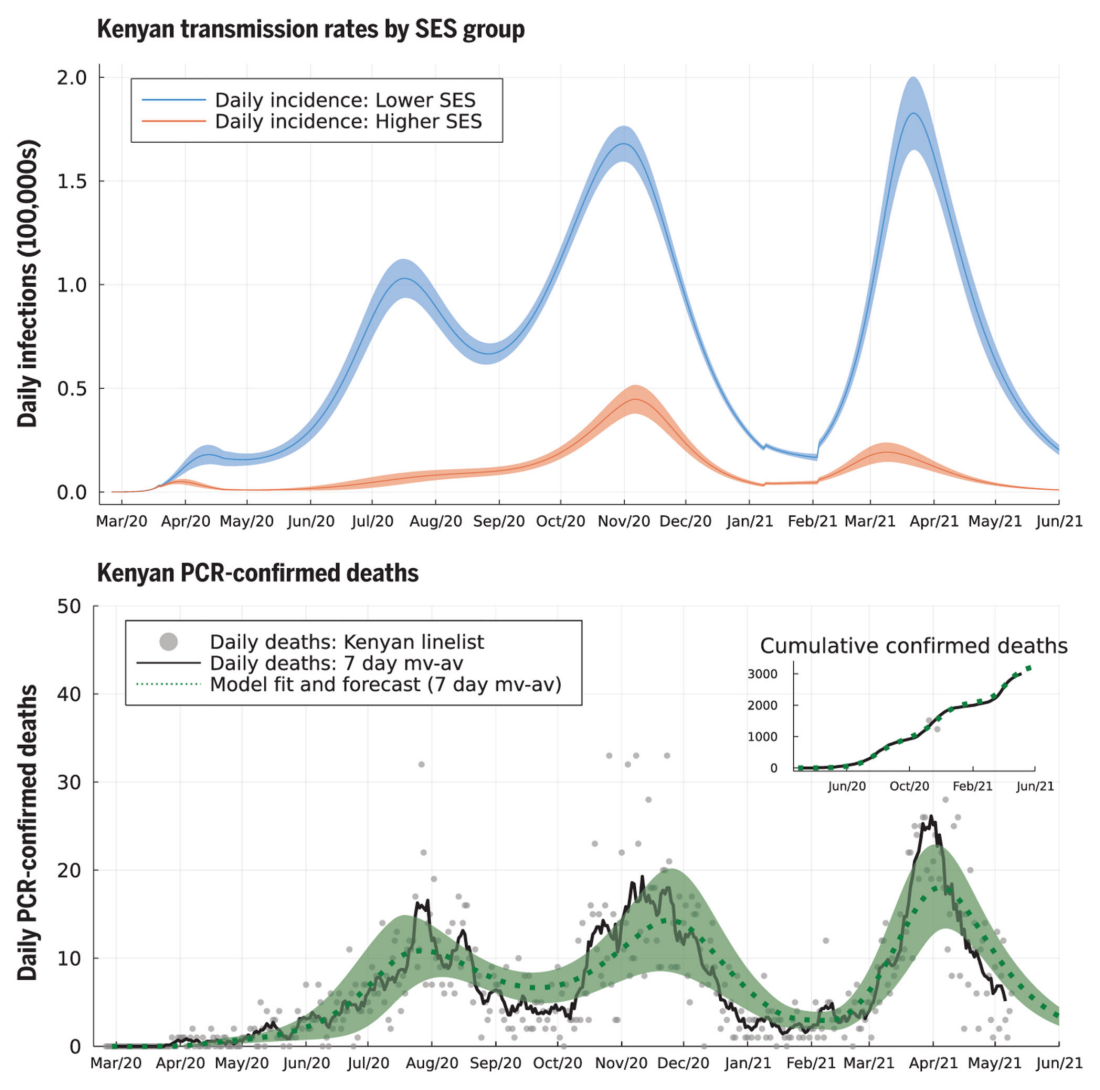
Model-inferred underlying true incidence rates by SES group relative to the whole Kenyan population size (top) and reported PCR-confirmed deaths due to COVID-19 disease (bottom). The size of the upper SES group was estimated to be 11% of the Kenyan population, explaining the lower absolute rate of incidence (red curve) compared to the lower SES group (blue curve). We inferred that the lower SES group has experienced three waves of SARS-CoV-2 transmission, whereas the upper SES group has experienced two. The model fit for 7-day moving average (green dashed curve, with shading as 95% prediction intervals) is shown against the 7-day moving average for deaths reported in the Kenyan linelist (black curve). Cumulative observed and fitted deaths are shown in the top-right inset.

## Data Availability

All code and data for the transmission model underlying this study is accessible at the Github repository (https://github.com/SamuelBrand1/kenya-covid-three-waves and at Zenodo (31). All data are available in the main text or the supplementary materials.
