# Supplementary material for: COVID-19 transmission dynamics underlying epidemic waves in Kenya
Source: Science. 2021 Oct 7;374(6570):989–94. doi: 10.1126/science.abk0414 (PMC7612211; doi:10.1126/science.abk0414)
Supplement: 20211007-1 [file science.abk0414.v1.pdf]

Cite as: S. P. C. Brand et al., Science
10.1126/science.abk0414 (2021).

COVID-19 transmission dynamics underlying epidemic waves in Kenya

Samuel P. C. Brand[1,2,3*†], John Ojal[1,4*†], Rabia Aziza[2,3], Vincent Were[5], Emelda A. Okiro[6,7], Ivy K Kombe[1], Caroline Mburu[1], Morris Ogero[1], Ambrose Agweyu[1,7], George M. Warimwe[1,7], James Nyagwange[1], Henry Karanja[1], John N. Gitonga[1], Daisy Mugo[1], Sophie Uyoga[1], Ifedayo M. O. Adetifa[1,8], J. Anthony G. Scott[1,8], Edward Otieno[1], Nickson Murunga[1], Mark Otiende[1], Lynette I. Ochola-Oyier[1], Charles N. Agoti[1], George Githinji[1], Kadondi Kasera[9], Patrick Amoth[9], Mercy Mwangangi[9], Rashid Aman[9], Wangari Ng'ang'a[10], Benjamin Tsofa[1], Philip Bejon[1,7], Matt. J. Keeling[2,3,11], D. James. Nokes[1,2,3], Edwine Barasa[5,7]

[1]Kenya Medical Research Institute (KEMRI) -Wellcome Trust Research Programme (KWTRP), Kilifi, Kenya. [2]The Zeeman Institute for Systems Biology and Infectious Disease Epidemiology Research (SBIDER), University of Warwick, Warwick, UK. [3]School of Life Sciences, University of Warwick, Warwick, UK. [4]London School of Hygiene and Tropical Medicine (LSHTM), London, UK. [5]Health Economics Research Unit, KEMRI-Wellcome Trust Research Programme, Nairobi, Kenya. [6]Population Health Unit, Kenya Medical Research Institute -Wellcome Trust Research Programme, Nairobi, Kenya. [7]Centre for Tropical Medicine and Global Health, Nuffield Department of Medicine, University of Oxford, Oxford, UK. [8]Department of Infectious Diseases Epidemiology, London School of Hygiene and Tropical Medicine, London, UK. [9]Ministry of Health, Government of Kenya, Nairobi, Kenya. [10]Presidential Policy and Strategy Unit, The Presidency, Government of Kenya. [11]Mathematics Institute, University of Warwick, Warwick, UK.

*Corresponding author. E-mail: s.brand@warwick.ac.uk; jojal@kemri-wellcome.org

†These authors contributed equally to this work.

Policy decisions on COVID-19 interventions should be informed by a local, regional and national understanding of SARS-CoV-2 transmission. Epidemic waves may result when restrictions are lifted or poorly adhered to, variants with new phenotypic properties successfully invade, or when infection spreads to susceptible sub-populations. Three COVID-19 epidemic waves have been observed in Kenya. Using a mechanistic mathematical model, we explain the first two distinct waves by differences in contact rates in high and low social-economic groups, and the third wave by the introduction of higher-transmissibility variants. Reopening schools led to a minor increase in transmission between the second and third waves. Socio-economic and urban/rural population structure are critical determinants of viral transmission in Kenya.

Following the first PCR confirmed case of COVID-19 in Kenya on 13th March 2020, the Kenyan government rapidly introduced measures aimed at suppressing SARS-CoV-2 transmission in the country. These measures included: the closure of international borders, with the exception of cargo movement; closing of schools and other learning institutions; a ban on social gatherings and meetings; closure of places of worship, bars and restaurants; a dawn to dusk curfew; mandatory wearing of masks in public places; physical distancing guidelines including on public transportation; and restrictions on movement into or out of counties with high infection rates including the two main Kenyan cities, Nairobi and Mombasa (*1*) (Fig. 1). Despite these measures the rate of new COVID-19 cases grew in Kenya indicating that measures had not been enough to consistently push the effective reproduction number $R(t) < 1$. Moreover, serological surveillance indicated that a higher than expected fraction of the Kenyan population had been exposed to SARS-CoV-2 given the case reports at the time: June 2020 adjusted seroprevalences, based on blood donor samples from the Kenya National Blood Transfusion Services (KNBTS), were 5.6% for Kenya, 8% for Mombasa, and 7.3% for Nairobi (*2*).

Detected COVID-19 incidence in Kenya first peaked in early August 2020 during a period of relaxation of measures: the end of the Nairobi and Coastal counties (including Mombasa) lockdown (7th June 2020), and the resumption of international air travel (1st August 2020). A single-wave epidemic in Kenya peaking within 100-200 days after SARS-CoV-2 introduction into the country was initially predicted, based on assumptions that included a single population group, and the development of immunity to reinfection (*3*–*6*). However, second and third waves occurred in mid-November 2020 and in March 2021, respectively. Multiple waves of COVID-19 incidence in High Income Country (HIC) settings have usually been associated with a relaxation of previous restrictions, for example in the UK (*7*). More recently, the emergence of new variants has been associated with further waves of infection (*8*). In Kenya, and other countries in Africa, a temporal association between relaxation of restrictions and subsequent waves is implausible. Understanding the causation of such multiple waves is critical for forecasting hospitalization demand and the likely effectiveness of interventions including

vaccination strategy.

There are multiple potential explanations for sequential wave dynamics in COVID-19 incidence, which might be acting singly or in concert: social clustering (*9*), changing adherence to measures over time (*7*), seasonal effects on transmission (*10*), re-opening of places of learning (*11*), lower transmission rates in rural settings leading to later peaks in those areas (*4*), waning immunity after an infection episode, as well as the introduction of new SARS-CoV-2 variants which are more transmissible than previous strains, or/and, evade prior immunity acquired by natural infection (*12*). The decrease in cases following the peak of the first wave occurred at a time of relaxation of social distancing measures in Kenya (Fig. 1). Hence, the end of the first wave cannot be explained by the imposition of non-pharmaceutical interventions. In this work, we present evidence that the most plausible explanation for the pattern of cases and seroprevalence observed in Kenya is a combination of differential adherence to measures between sub-populations which we identify with lower and higher socio-economic status (SES) in 2020 followed by a sharp increase in virus transmissibility in 2021, consistent with that observed in other countries affected by variants of concern, e.g., the United Kingdom (*13*) and India (*14*). Previous studies undertaken in sub-Saharan Africa at the level of individual country (*4*) or pan-African exploring the impact of climate (*15*) have not had the opportunity to integrate longitudinal PCR, serology and Google mobility data.

We developed a county-specific, two-socio-economic status (SES) group, SEIRS-type transmission model, using a waning immunity rate derived from recent studies on the protectiveness of a natural infection to future reinfection (*16*–*19*). Our model includes, for each Kenyan county: a) a SEIRS transmission model predicting new infections on each day, socio-economic group, and county, which accounts for assortativity in infections; that is the propensity for infected individuals to cause more intra-group infections compared to inter-group infections; and b) an observation model reflecting the data streams: PCR testing (positive and negative results), seroprevalence surveys, google mobility data, and determined COVID-19 deaths. The model developed for this paper differs from the standard SEIRS model with homogeneous mixing, adding the impact of new variants as detected by genomic surveillance and allowing the model to fit two socioeconomic groups in counties where this was supported by the data streams. We used a hierarchical approach to inferring the underlying epidemic trajectories in each of the 47 Kenyan semi-autonomous counties by the following three steps: a) grouping counties by similarity over a range of sociological and epidemiological metrics using machine learning; b) for the 11 counties with a relatively high density of serology tests we jointly inferred epidemiological model parameters for each county e.g., i) baseline R_0 for the county, ii) the effect of schools being open on R(t), iii) the increase in transmissibility in February 2021 when B.1.1.7 lineage (alpha variant) SARS-CoV-2 was first detected in Kenya (*20*), iv) the fraction of the population in the higher SES group in each county and their assortative mixing rate, and v) the fraction of cases reported for the county using Hamiltonian Markov chain Monte Carlo (*21*) with mildly informative priors, and c) we inferred model parameters for the remaining 36 counties using informative priors for reporting fractions derived from a synthesis of the posterior distributions of counties grouped as similar to that county (see supplementary materials for details). We conducted formal model selection to compare one, two, and three socio-economic status group models, finding that the one-group model was an inadequate fit to the data, and the three-group model was not an improvement on the two-group model (see supplementary materials). We have also conducted sensitivity analysis for different assumptions on waning immunity, finding consistent results for a range of scenarios (see supplementary materials).

The two-SES group transmission model was able to capture the timing and intensity of all three waves of Kenyan COVID case incidence and the trend of increasing proportion seropositive among KNBTS donors (Fig. 2). We also validated the fitted model by comparing forecasts of seropositivity rates with those from data not used to infer model parameters. We used rounds 1 and 2 of the seropositivity survey using KNBTS donors for model parameter inference, collected during May – September 2020. Estimated seroprevalence among the Kenyan population, derived from the fitted two-SES group transmission model, was in good agreement with the out-of-sample round 3 of KNBTS seroprevalence data, collected January – March 2021 (Fig. 2). The Nairobi-specific epidemic trajectory inferred in this study agrees with seroprevalence estimates from a randomized survey from Nairobi, and, is congruent with the observation that it was public hospitals in Nairobi (favored by lower SES groups) that came under pressure in the first wave, whereas the second wave showed increased admission to private health facilities (figs. S7 and S8). As well as capturing the past trends of case reporting and seropositivity in Kenya, the fitted two-SES group transmission model accurately predicts the daily rate of new confirmed COVID-19 cases reported by the Kenyan Ministry of Health for the month after the censoring date of the PCR test data used to infer model parameters (Fig. 2).

The two-SES group transmission model reconciled the apparent paradox between evidence of the effectiveness of the rapidly introduced Kenyan measures in reducing mobility out of the home among Kenyan smartphone users, which was close to that observed in European and North American countries (fig. S1), and that case rates and fatality rates display two distinct waves in Kenya in 2020. The model provides

an explanation for the end of first wave through the depletion of susceptibles in geographically distinct, largely urban, subpopulations of lower socio-economic status. In some Kenyan counties (e.g., the urban counties Nairobi and Mombasa, and some of the semi-urban counties) we infer that a substantial group of people belong to the higher SES group whose mobility is well-represented by Google smartphone data; a combination of school closures and reduction in mobility (by 44.5% see supplementary materials) reduced the effective reproductive number sufficiently that newly infected people among the higher SES group were on average generating less than 1 secondary infection by April 2020 (Fig. 3). In the counties where the model finds evidence for distinct two group dynamics (fig. S13), the model predicts low rates of inter-group infection transmission (posterior mean for the assortativity parameter estimates of 2-40 disassortative infections per 1000 potential infection events). We believe this can be ascribed to pandemic-induced changes in social behavior that enhanced intra-SES group infectious contacts (such as longer contact durations in families or local communities) and decreased inter-SES group infectious contacts due to, for example, avoiding public transport and cancelling domestic staff visits. The growth rate in cases, and relatively high levels of seroprevalence among KNBTS donors, are explained by the rest of the population in the lower SES group having R(t) > 1 into May and June 2020 (Fig. 3). The model inferred that the reduction in mobility among the lower SES group was substantially less than among the higher SES group: the posterior mean estimate for reduction in mobility among the lower SES group in Nairobi was 13.8% (CI 11.3-17.5%), in Kenya's second city Mombasa was 18.9% (CI 17.4-20.4%), and posterior mean estimates for lower SES group mobility reduction across all 47 Kenyan counties had a median of 15.7% (IQR 10.9-19.6%). We assumed that school closures reduced R(t) for both SES groups equally. The inferred reduction in R(t) due to schools closing varied from county to county, the median reduction in R(t) over counties was 13.5% (IQR 4.3-23.7%; Nairobi estimate for school closure effect was 23.8% CI 16.5-31.6%, Mombasa estimate for school closure effect was 20.2% CI 15.2-25.2%; Fig. 3).

The second wave in Kenya in 2020 was due to the superimposition of two trends: a) in mainly urban areas, a second wave was triggered by the higher SES group returning to pre-COVID-19 mobility patterns by early November 2020 (fig. S1), and, therefore, R(t) was >1 for the higher SES group (Fig. 3 top and bottom left); and b) in more rural areas the inferred size of the higher SES group was small, and R(t) was low but persistently >1 for the lower SES group (R(t) ~ 1-1.5) until November 2020 (Fig. 3 bottom right, fig. S13, and see supplementary materials). Low rates of mobility somewhat shielded the higher SES group from infection in the first wave among the lower SES group. Therefore, the lower SES group, in cities, suffered two waves in 2020, whereas the higher SES group effectively suffered one wave peaking in late 2020 (Fig. 4). The overall detection rate was determined in part by the number of PCR tests performed each day, and this rate dropped in September 2020 (fig. S4). A consequence of the drop in the testing rate was that the case reporting shows a much sharper delineation between the first two waves (Fig. 2) than the underlying inferred infection rates (Fig. 4), which reveal that there was only a moderate dip in infections in August/September 2020. By accounting for the delay between infection and COVID-19 fatality, and fitting SES group specific infection-fatality-detection ratios (IFR-detection, see materials and methods and supplementary materials) to each county, we found reasonable agreement between the predicted and observed timings of peak fatality rates in Kenya (Fig. 4). Overall, our model-based estimate was that only 11% of the total Kenyan population were in the higher SES group, whose mobility was well-described by Google mobility data, with the highest concentration of higher SES group individuals in the two main cities: 43.4% of the Nairobi population (CI 35.4-49.2%) and 40.3% of the Mombasa population (CI 35.0-45.4%). Additionally, we estimate that infections among the higher SES group were substantially more likely to be detected than among the lower SES group: odds ratio for Nairobi for detection per case between higher and lower SES 4.5 (CI 1.5-17.9), for Mombasa for detection per case between higher and lower SES 4.8 (CI 3.2-6.8). The odds ratio between detection per infection in the two SES groups was inferred to be even more extreme across Kenya as a whole, with substantial variation from county to county: median odds ratio estimate over counties was 18.5 (counties estimate IQR 2.5-27.9), although most counties had a small number of people in the higher SES group.

Fully reopening schools in January 2021 was associated with a slight increase in cases and deaths in Kenya, with a peak in January and early February 2021 (Figs. 3 and 4). However, school reopening does not explain the third wave in Kenya observed in March and April 2021, which was considerably larger than the increase in January/February 2021. The two SES group model was not a sufficient explanation for a third wave, neither was loss of immunity or any detectable trend in mobility. The first cases of the more transmissible Alpha variant B.1.1.7 were identified in Kenya from mid-January 2021 (*20*). We investigated if the data supported an increase in transmissibility per infected person starting at the beginning of February 2021 as well as an influx of new exposed individuals representing domination of wildtype strains of SARS-CoV-2 by a fitter new variant. In the Kenyan urban counties, we found credible range of increase in transmissibility of 15.0-46.6% (Nairobi 32.5% CI 18.1-46.6%; Mombasa 22.8% CI 15.0-31.2%), and this was reflected in increased transmissibility estimates across Kenyan counties: median

over county estimates 46.1% (IQR 31.6-72.9%). The fitted model predicted that this large increase in transmissibility will push the overall exposure to SARS-CoV-2 in Kenya from a back-calculated estimate of 53.5% in February 2021 to 78.1% by June 2021 (Fig. 2). The rate of seroreversion, that is the loss of detectable antibodies rather than necessarily loss of protective immunity, has been identified as an important quantity for estimating population exposure prevalence from serological data (*22*). Because the serological data used for parameter inference was collected within 7 months of the first identified case in Kenya, we assumed that seroreversion was negligible over this period. However, we note that assuming no future seroreversion led to closer agreement between model back-calculation and round 3 KNBTS data than assuming a median 1 year between infection and seroreversion (Fig. 2); that is that our modelling doesn't support the need to incorporate seroreversion to capture the true population exposure over the time scale of a year, unlike for Buss *et al.*_ (*22*). This finding highlights that_seroreversion rate depends on the serological assay used (*23*) and cannot necessarily be extrapolated between settings. A negligible seroreversion rate may be more applicable for the ELISA used in Kenya where the cut-offs prioritize specificity over sensitivity (*2*, *24*).

Our modelling exercise provides a credible mechanistic interpretation of the three waves of COVID-19 in Kenya. We hypothesize the presence of two SES groups in each county and allow the model freedom to fit the relative proportion in each by county, inferred from locally collected PCR and serological test data. The model results support the notion of two SES groups in urban settings defined by highly assortative mixing (Nairobi, Mombasa and predominantly counties near Nairobi) whereas for most rural counties mixing was inferred to be less assortative and/or effectively all the population is in a single SES group (fig. S13). We invoke two key underlying assumptions. First, a stratified population differing in mobility (associated with lower and higher SES), and second, increased virus transmissibility compatible with competitive succession of a SARS-CoV-2 variant of concern in wave 3. A key simplifying assumption in this modelling study is that we assumed that the diversity of behaviors across the population in each Kenyan county can be reduced to stratifying into two groups with assortative mixing favoring transmission within group, and identifying these groups into lower and higher SES groups based on similarity to mobility trends among smart phone users. We believe that this is a well-evidenced hypothesis for Kenya. Marked social and economic structuring has been described in Kenya; 36% of the population live below the national poverty line (*25*) and 55% live in informal settlements (*26*). Further, 83% of Kenya's labor market is informal, characterized by unstable and unpredictable daily wages (*27*). Lower socio-economic groups have been identified as vulnerable to SARS-CoV-2 in the global South due to residence in informal settlements at high population density, reduced access to sanitation, and dependence on informal employment requiring daily mobility (*28*, *29*). In contrast, the higher SES group with job security can work from home, socially distance and readily access water and sanitation, thereby decreasing transmission. In Kenya, Google mobility data from smartphone users indicates a sharp decline in movement to settings outside of the home (fig. S1). We found that the two SES group model used in this paper was able to capture pattern of cases and seroprevalence in Kenya over the first three waves, despite radically simplifying the underlying social structure.

We predict the proportion of the Kenya population exposed to SARS-CoV-2 to be greater than 75% by the beginning of June 2021 (Fig. 2), corresponding to around 39 million people. However, less than 4,000 confirmed COVID-19 deaths and 180,000 confirmed SARS-CoV-2 infections have been identified as of the 1st June 2021. We found that people among the lower SES group were likely to be even more under-sampled than people among the upper SES group, as well as identifying wide regional variation in the detection rate. These results emphasize the necessity of community surveys of SARS-CoV-2 prevalence, exposure, and an investigation into the under-reporting of mortality and severe disease due to COVID-19. Multiple waves have been observed in many other African countries that do not appear to be completely explained by the timing of restrictions, and since they also have in common similar socio-economic groupings in urban centres, we speculate the explanations found to be plausible in our model for Kenya may apply more widely.

The high population exposure suggests that a fourth COVID-19 wave in Kenya before the end of 2021 would only be likely if (i) a variant arises with substantial further enhancement in transmissibility or immune escape, such as the B.1.617.2 Delta variant (*30*), or (ii) significant waning of immunity in those previously exposed. We predict that approximately 75% of the Kenyan population have been exposed to SARS-CoV-2 by June 2021. This will mitigate the death rate that might be expected in the future but taking together a) the markedly increased transmissibility of Delta variant; b) the potential for re-infection and c) the experience of other countries despite prevalent vaccination, this scenario is entirely consistent with a significant fourth wave in Kenya. We conclude that our analysis which triangulates PCR test, seroprevalence, mobility and genomic data to develop a coherent explanation of the transmission dynamics of COVID-19, provides insight of public health importance in Kenya, including targeting health care capacity and pharmaceutical and non-pharmaceutical interventions.

REFERENCES AND NOTES

1. E. Barasa *et al*., "Assessing the Indirect Health Effects of the COVID-19 Pandemic in Kenya" (2021); https://www.cgdev.org/publication/assessing-indirect-health-

effects-covid-19-pandemic-kenya.
2. S. Uyoga, I. M. O. Adetifa, H. K. Karanja, J. Nyagwange, J. Tuju, P. Wanjiku, R. Aman, M. Mwangangi, P. Amoth, K. Kasera, W. Ng'ang'a, C. Rombo, C. Yegon, K. Kithi, E. Odhiambo, T. Rotich, I. Orgut, S. Kihara, M. Otiende, C. Bottomley, Z. N. Mupe, E. W. Kagucia, K. E. Gallagher, A. Etyang, S. Voller, J. N. Gitonga, D. Mugo, C. N. Agoti, E. Otieno, L. Ndwiga, T. Lambe, D. Wright, E. Barasa, B. Tsofa, P. Bejon, L. I. Ochola-Oyier, A. Agweyu, J. A. G. Scott, G. M. Warimwe, Seroprevalence of anti-SARS-CoV-2 IgG antibodies in Kenyan blood donors. *Science* **371**, 79–82 (2021). doi:10.1126/science.abe1916 Medline
3. S. P. C. Brand *et al*., Forecasting the scale of the COVID-19 epidemic in Kenya. *medRxiv* 2020.04.09.20059865 [Preprint]. 16 April 2020. https://doi.org/10.1101/2020.04.09.20059865.
4. B. Z. Diop, M. Ngom, C. Pougué Biyong, J. N. Pougué Biyong, The relatively young and rural population may limit the spread and severity of COVID-19 in Africa: A modelling study. *BMJ Glob. Health* **5**, e002699–e11 (2020). doi:10.1136/bmjgh-2020-002699 Medline
5. S. Mwalili, M. Kimathi, V. N. Ojiambo, D. K. Gathungu, T. N. O. Achia, Age-structured Impact of Mitigation Strategies on COVID-19 Severity and Deaths in Kenya. *ResearchSquare* (2020), doi:10.21203/rs.3.rs-105797/v1.
6. J. Ojal, S. P. C. Brand, M. J. Keeling, D. James Nokes, Revealing the extent of the first wave of the COVID-19 pandemic in Kenya based on serological and PCR-test data. *Wellcome Open Res.* **6**, 127 (2021). doi:10.12688/wellcomeopenres.16748.1
7. M. J. Keeling, E. M. Hill, E. E. Gorsich, B. Penman, G. Guyver-Fletcher, A. Holmes, T. Leng, H. McKimm, M. Tamborrino, L. Dyson, M. J. Tildesley, Predictions of COVID-19 dynamics in the UK: Short-term forecasting and analysis of potential exit strategies. *PLOS Comput. Biol.* **17**, e1008619–e1008620 (2021). doi:10.1371/journal.pcbi.1008619 Medline
8. H. Tegally, E. Wilkinson, M. Giovanetti, A. Iranzadeh, V. Fonseca, J. Giandhari, D. Doolabh, S. Pillay, E. J. San, N. Msomi, K. Mlisana, A. von Gottberg, S. Walaza, M. Allam, A. Ismail, T. Mohale, A. J. Glass, S. Engelbrecht, G. Van Zyl, W. Preiser, F. Petruccione, A. Sigal, D. Hardie, G. Marais, N. Y. Hsiao, S. Korsman, M. A. Davies, L. Tyers, I. Mudau, D. York, C. Maslo, D. Goedhals, S. Abrahams, O. Laguda-Akingba, A. Alisoltani-Dehkordi, A. Godzik, C. K. Wibmer, B. T. Sewell, J. Lourenço, L. C. J. Alcantara, S. L. Kosakovsky Pond, S. Weaver, D. Martin, R. J. Lessells, J. N. Bhiman, C. Williamson, T. de Oliveira, Detection of a SARS-CoV-2 variant of concern in South Africa. *Nature* **592**, 438–443 (2021). Medline
9. B. F. Nielsen, L. Simonsen, K. Sneppen, COVID-19 Superspreading Suggests Mitigation by Social Network Modulation, COVID-19 Superspreading Suggests Mitigation by Social Network Modulation. *Phys. Rev. Lett.* **126**, 118301 (2021). doi:10.1103/PhysRevLett.126.118301 Medline
10. S. M. Kissler, C. Tedijanto, E. Goldstein, Y. H. Grad, M. Lipsitch, Projecting the transmission dynamics of SARS-CoV-2 through the postpandemic period. *Science* **368**, 860–868 (2020). doi:10.1126/science.abb5793 Medline
11. J. Panovska-Griffiths, C. C. Kerr, R. M. Stuart, D. Mistry, D. J. Klein, R. M. Viner, C. Bonell, Determining the optimal strategy for reopening schools, the impact of test and trace interventions, and the risk of occurrence of a second COVID-19 epidemic wave in the UK: A modelling study. *Lancet Child Adolesc. Health* **4**, 817–827 (2020). Medline
12. T. Kirby, New variant of SARS-CoV-2 in UK causes surge of COVID-19. *Lancet Respir. Med.* **9**, e20–e21 (2021). doi:10.1016/S2213-2600(21)00005-9 Medline
13. N. G. Davies, S. Abbott, R. C. Barnard, C. I. Jarvis, A. J. Kucharski, J. D. Munday, C. A. B. Pearson, T. W. Russell, D. C. Tully, A. D. Washburne, T. Wenseleers, A. Gimma, W. Waites, K. L. M. Wong, K. van Zandvoort, J. D. Silverman, K. Diaz-Ordaz, R. Keogh, R. M. Eggo, S. Funk, M. Jit, K. E. Atkins, W. J. Edmunds; CMMID COVID-19 Working Group; COVID-19 Genomics UK (COG-UK) Consortium, Estimated transmissibility and impact of SARS-CoV-2 lineage B.1.1.7 in England. *Science* **372**, eabg3055 (2021). doi:10.1126/science.abg3055 Medline
14. J. Singh, S. A. Rahman, N. Z. Ehtesham, S. Hira, S. E. Hasnain, SARS-CoV-2 variants of concern are emerging in India. *Nat. Med.* **27**, 1131–1133 (2021). Medline
15. R. E. Baker, W. Yang, G. A. Vecchi, C. J. E. Metcalf, B. T. Grenfell, Susceptible supply limits the role of climate in the early SARS-CoV-2 pandemic. *Science* **369**, 315–319 (2020). doi:10.1126/science.abc2535 Medline
16. R. A. Harvey, J. A. Rassen, C. A. Kabelac, W. Turenne, S. Leonard, R. Klesh, W. A. Meyer 3rd, H. W. Kaufman, S. Anderson, O. Cohen, V. I. Petkov, K. A. Cronin, A. L. Van Dyke, D. R. Lowy, N. E. Sharpless, L. T. Penberthy, Association of SARS-CoV-2 seropositive antibody test with risk of future infection. *JAMA Intern. Med.* **181**, 672–679 (2021). doi:10.1001/jamainternmed.2021.0366 Medline
17. V. J. Hall, S. Foulkes, A. Charlett, A. Atti, E. J. M. Monk, R. Simmons, E. Wellington, M. J. Cole, A. Saei, B. Oguti, K. Munro, S. Wallace, P. D. Kirwan, M. Shrotri, A. Vusirikala, S. Rokadiya, M. Kall, M. Zambon, M. Ramsay, T. Brooks, C. S. Brown, M. A. Chand, S. Hopkins, N. Andrews, A. Atti, H. Aziz, T. Brooks, C. S. Brown, D. Camero, C. Carr, M. A. Chand, A. Charlett, H. Crawford, M. Cole, J. Conneely, S. D'Arcangelo, J. Ellis, S. Evans, S. Foulkes, N. Gillson, R. Gopal, L. Hall, V. J. Hall, P. Harrington, S. Hopkins, J. Hewson, K. Hoschler, D. Ironmonger, J. Islam, M. Kall, I. Karagiannis, O. Kay, J. Khawam, E. King, P. Kirwan, R. Kyffin, A. Lackenby, M. Lattimore, E. Linley, J. Lopez-Bernal, L. Mabey, R. McGregor, S. Miah, E. J. M. Monk, K. Munro, Z. Naheed, A. Nissr, A. M. O'Connell, B. Oguti, H. Okafor, S. Organ, J. Osbourne, A. Otter, M. Patel, S. Platt, D. Pople, K. Potts, M. Ramsay, J. Robotham, S. Rokadiya, C. Rowe, A. Saei, G. Sebbage, A. Semper, M. Shrotri, R. Simmons, A. Soriano, P. Staves, S. Taylor, A. Taylor, A. Tengbe, S. Tonge, A. Vusirikala, S. Wallace, E. Wellington, M. Zambon, D. Corrigan, M. Sartaj, L. Cromey, S. Campbell, K. Braithwaite, L. Price, L. Haahr, S. Stewart, E. D. Lacey, L. Partridge, G. Stevens, Y. Ellis, H. Hodgson, C. Norman, B. Larru, S. Mcwilliam, S. Winchester, P. Cieciwa, A. Pai, C. Loughrey, A. Watt, F. Adair, A. Hawkins, A. Grant, R. Temple-Purcell, J. Howard, N. Slawson, C. Subudhi, S. Davies, A. Bexley, R. Penn, N. Wong, G. Boyd, A. Rajgopal, A. Arenas-Pinto, R. Matthews, A. Whileman, R. Laugharne, J. Ledger, T. Barnes, C. Jones, D. Botes, N. Chitalia, S. Akhtar, G. Harrison, S. Horne, N. Walker, K. Agwuh, V. Maxwell, J. Graves, S. Williams, A. O'Kelly, P. Ridley, A. Cowley, H. Johnstone, P. Swift, J. Democratis, M. Meda, C. Callens, S. Beazer, S. Hams, V. Irvine, B. Chandrasekaran, C. Forsyth, J. Radmore, C. Thomas, K. Brown, S. Roberts, P. Burns, K. Gajee, T. M. Byrne, F. Sanderson, S. Knight, E. Macnaughton, B. J. L. Burton, H. Smith, R. Chaudhuri, K. Hollinshead, R. J. Shorten, A. Swan, R. J. Shorten, C. Favager, J. Murira, S. Baillon, S. Hamer, K. Gantert, J. Russell, D. Brennan, A. Dave, A. Chawla, F. Westell, D. Adeboyeku, P. Papineni, C. Pegg, M. Williams, S. Ahmad, S. Ingram, C. Gabriel, K. Pagget, P. Cieciwa, G. Maloney, J. Ashcroft, I. Del Rosario, R. Crosby-Nwaobi, C. Reeks, S. Fowler, L. Prentice, M. Spears, G. McKerron, K. McLelland-Brooks, J. Anderson, S. Donaldson, K. Templeton, L. Coke, N. Elumogo, J. Elliott, D. Padgett, M. Mirfenderesky, A. Cross, J. Price, S. Joyce, I. Sinanovic, M. Howard, T. Lewis, P. Cowling, D. Potoczna, S. Brand, L. Sheridan, B. Wadams, A. Lloyd, J. Mouland, J. Giles, G. Pottinger, H. Coles, M. Joseph, M. Lee, S. Orr, H. Chenoweth, C. Auckland, R. Lear, T. Mahungu, A. Rodger, K. Penny-Thomas, S. Pai, J. Zamikula, E. Smith, S. Stone, E. Boldock, D. Howcroft, C. Thompson, M. Aga, P. Domingos, S. Gormley, C. Kerrison, L. Marsh, S. Tazzyman, L. Allsop, S. Ambalkar, M. Beekes, S. Jose, J. Tomlinson, A. Jones, C. Price, J. Pepperell, M. Schultz, J. Day, A. Boulos, E. Defever, D. McCracken, K. Brown, K. Gray, A. Houston, T. Planche, R. Pritchard Jones, D. Wycherley, S. Bennett, J. Marrs, K. Nimako, B. Stewart, N. Kalakonda, S. Khanduri, A. Ashby, M. Holden, N. Mahabir, J. Harwood, B. Payne, K. Court, N. Staines, R. Longfellow, M. E. Green, L. E. Hughes, M. Halkes, P. Mercer, A. Roebuck, E. Wilson-Davies, L. Gallego, R. Lazarus, N. Aldridge, L. Berry, F. Game, T. Reynolds, C. Holmes, M. Wiselka, A. Higham, M. Booth, C. Duff, J. Alderton, H. Jory, E. Virgilio, T. Chin, M. Z. Qazzafi, A. M. Moody, R. Tilley, T. Donaghy, K. Shipman, R. Sierra, N. Jones, G. Mills, D. Harvey, Y. W. J. Huang, J. Birch, L. Robinson, S. Board, A. Broadley, C. Laven, N. Todd, D. W. Eyre, K. Jeffery, S. Dunachie, C. Duncan, P. Klenerman, L. Turtle, T. De Silva, H. Baxendale, J. L. Heeney; SIREN Study Group, SARS-CoV-2 infection rates of antibody-positive compared with antibody-negative health-care workers in England: A large, multicentre, prospective cohort study (SIREN). *Lancet* **397**, 1459–1469 (2021). doi:10.1016/S0140-6736(21)00675-9 Medline
18. J. Vitale, N. Mumoli, P. Clerici, M. De Paschale, I. Evangelista, M. Cei, A. Mazzone, Assessment of SARS-CoV-2 Reinfection 1 Year After Primary Infection in a Population in Lombardy, Italy. *JAMA Intern. Med.* **181**, 1407–1408 (2021). doi:10.1001/jamainternmed.2021.2959 Medline
19. F. Lumley, J. Wei, D. O'Donnell, N. E. Stoesser, P. C. Matthews, A. Howarth, S. B. Hatch, B. D. Marsden, S. Cox, T. James, L. J. Peck, T. G. Ritter, Z. de Toledo, R. J. Cornall, E. Y. Jones, D. I. Stuart, G. Screaton, D. Ebner, S. Hoosdally, D. W. Crook, C. P. Conlon, K. B. Pouwels, A. S. Walker, T. E. A. Peto, T. M. Walker, K. Jeffery, D. W. Eyre; Oxford University Hospitals Staff Testing Group, The Duration, Dynamics, and Determinants of Severe Acute Respiratory Syndrome Coronavirus 2 (SARS-CoV-2) Antibody Responses in Individual Healthcare Workers. *Clin. Infect. Dis.* **73**,

e699–e709 (2021). doi:10.1093/cid/ciab004 Medline
20. E. Wilkinson, M. Giovanetti, H. Tegally, J. E. San, E. T. Cirulli, A year of genomic surveillance reveals how the SARS-CoV-2 pandemic unfolded in Africa. *medRxiv* 2021.05.12.21257080 [Preprint]. 13 May 2021. https://doi.org/10.1101/2021.05.12.21257080.
21. A. Chatzilena, E. van Leeuwen, O. Ratmann, M. Baguelin, N. Demiris, Contemporary statistical inference for infectious disease models using Stan. *Epidemics* **29**, 100367 (2019). doi:10.1016/j.epidem.2019.100367 Medline
22. L. F. Buss, C. A. Prete Jr., C. M. M. Abrahim, A. Mendrone Jr., T. Salomon, C. de Almeida-Neto, R. F. O. França, M. C. Belotti, M. P. S. S. Carvalho, A. G. Costa, M. A. E. Crispim, S. C. Ferreira, N. A. Fraiji, S. Gurzenda, C. Whittaker, L. T. Kamaura, P. L. Takecian, P. da Silva Peixoto, M. K. Oikawa, A. S. Nishiya, V. Rocha, N. A. Salles, A. A. de Souza Santos, M. A. da Silva, B. Custer, K. V. Parag, M. Barral-Netto, M. U. G. Kraemer, R. H. M. Pereira, O. G. Pybus, M. P. Busch, M. C. Castro, C. Dye, V. H. Nascimento, N. R. Faria, E. C. Sabino, Three-quarters attack rate of SARS-CoV-2 in the Brazilian Amazon during a largely unmitigated epidemic. *Science* **371**, 288–292 (2021). doi:10.1126/science.abe9728 Medline
23. The COVID-19 Community Research Partnership Study Group, Duration of SARS-CoV-2 Sero-Positivity in a Large Longitudinal Sero-Surveillance Cohort: The COVID-19 Community Research Partnership. *medRxiv* 2021.01.27.21250615 [Preprint]. 29 January 2021. https://doi.org/10.1101/2021.01.27.21250615
24. C. Bottomley *et al*., Improving SARS-CoV-2 cumulative incidence estimation through mixture modelling of antibody levels. *medRxiv* 2021.04.09.21254250 [Preprint]. 13 April 2021. https://doi.org/10.1101/2021.04.09.21254250.
25. Kenyan National Bureau of Statistics Ministry Of Devolution National Planning, "KENYA - Kenya Integrated Household Budget Survey 2015-2016" (2018).
26. UN-Habitat, "UN-Habitat Support to Sustainable Urban Development in Kenya" (2017).
27. The World Bank Group, "Kenya Economic Update" (2016).
28. P. M. Macharia, N. K. Joseph, E. A. Okiro, A vulnerability index for COVID-19: spatial analysis to inform equitable response in Kenya. *medRxiv* 2020.05.27.20113803 [Preprint]. 28 May 2020. https://doi.org/10.1101/2020.05.27.20113803.
29. J. Corburn, D. Vlahov, B. Mberu, L. Riley, W. T. Caiaffa, S. F. Rashid, A. Ko, S. Patel, S. Jukur, E. Martínez-Herrera, S. Jayasinghe, S. Agarwal, B. Nguendo-Yongsi, J. Weru, S. Ouma, K. Edmundo, T. Oni, H. Ayad, Slum Health: Arresting COVID-19 and Improving Well-Being in Urban Informal Settlements. *J. Urban Health* **97**, 348–357 (2020). doi:10.1007/s11524-020-00438-6 Medline
30. I. Ferreira *et al*., SARS-CoV-2 B.1.617 emergence and sensitivity to vaccine-elicited antibodies. *bioRxiv* 2021.05.08.443253 [Preprint]. 9 May 2021. https://doi.org/10.1101/2021.05.08.443253.
31. S. Brand *et al*., SamuelBrand1/kenya-covid-three-waves: This is the version of the KenyaCoVSD module associated with the paper "COVID-19 Transmission Dynamics Underlying Epidemic Waves in Kenya," (Version KenyaCoVSDv1), Zenodo (2021); https://doi.org/10.5281/zenodo.5541433.
32. L. L. C. Google, *Google COVID-19 Community Mobility Reports*; https://www.google.com/covid19/mobility/.
33. S. Chang, E. Pierson, P. W. Koh, J. Gerardin, B. Redbird, D. Grusky, J. Leskovec, Mobility network models of COVID-19 explain inequities and inform reopening. *Nature* **589**, 82–87 (2021). Medline
34. Technology use in Africa: Smartphones (2018); https://www.pewresearch.org/global/interactives/technology-use-in-africa-smartphones/.
35. M. J. Keeling, P. Rohani, Estimating spatial coupling in epidemiological systems: A mechanistic approach. *Ecol. Lett.* **5**, 20–29 (2002). doi:10.1046/j.1461-0248.2002.00268.x
36. M. J. Keeling *et al*., "Metapopulation dynamics of infectious diseases" in *Ecology, Genetics and Evolution of Metapopulations* (Academic Press, 2004), pp. 415–445.
37. M. J. Keeling, P. Rohani, *Modeling Infectious Diseases in Humans and Animals* (Princeton Univ. Press, 2008).
38. L. Ferretti, C. Wymant, M. Kendall, L. Zhao, A. Nurtay, L. Abeler-Dörner, M. Parker, D. Bonsall, C. Fraser, Quantifying SARS-CoV-2 transmission suggests epidemic control with digital contact tracing. *Science* **368**, eabb6936–9 (2020). doi:10.1126/science.abb6936 Medline
39. J. Wallinga, M. Lipsitch, How generation intervals shape the relationship between growth rates and reproductive numbers. *Proc. Biol. Sci.* **274**, 599–604 (2007). Medline
40 S. A. Lauer, K. H. Grantz, Q. Bi, F. K. Jones, Q. Zheng, H. R. Meredith, A. S. Azman, N. G. Reich, J. Lessler, The Incubation Period of Coronavirus Disease 2019 (COVID-19) From Publicly Reported Confirmed Cases: Estimation and Application. *Ann. Intern. Med.* **172**, 577–582 (2020). doi:10.7326/M20-0504 Medline
41. L. C. Tindale, J. E. Stockdale, M. Coombe, E. S. Garlock, W. Y. V. Lau, M. Saraswat, L. Zhang, D. Chen, J. Wallinga, C. Colijn, Evidence for transmission of COVID-19 prior to symptom onset. *eLife* **9**, e57149 (2020). doi:10.7554/eLife.57149 Medline
42. A. Gelman *et al*., *Bayesian Data Analysis* (CRC Press, 2013).
43. F. Zhou, T. Yu, R. Du, G. Fan, Y. Liu, Z. Liu, J. Xiang, Y. Wang, B. Song, X. Gu, L. Guan, Y. Wei, H. Li, X. Wu, J. Xu, S. Tu, Y. Zhang, H. Chen, B. Cao, Clinical course and risk factors for mortality of adult inpatients with COVID-19 in Wuhan, China: A retrospective cohort study. *Lancet* **395**, 1054–1062 (2020). doi:10.1016/S0140-6736(20)30566-3 Medline
44. M. Lisboa Bastos, G. Tavaziva, S. K. Abidi, J. R. Campbell, L.-P. Haraoui, J. C. Johnston, Z. Lan, S. Law, E. MacLean, A. Trajman, D. Menzies, A. Benedetti, F. Ahmad Khan, Diagnostic accuracy of serological tests for covid-19: Systematic review and meta-analysis. *BMJ* **370**, m2516–m13 (2020). doi:10.1136/bmj.m2516 Medline
45. C. Rackauckas, Q. Nie, DifferentialEquations. jl–A Performant and Feature-Rich Ecosystem for Solving Differential Equations in Julia. *J. Open Res. Softw.* **5**, 15 (2017). doi:10.5334/jors.151
46. R. Verity, L. C. Okell, I. Dorigatti, P. Winskill, C. Whittaker, N. Imai, G. Cuomo-Dannenburg, H. Thompson, P. G. T. Walker, H. Fu, A. Dighe, J. T. Griffin, M. Baguelin, S. Bhatia, A. Boonyasiri, A. Cori, Z. Cucunubá, R. FitzJohn, K. Gaythorpe, W. Green, A. Hamlet, W. Hinsley, D. Laydon, G. Nedjati-Gilani, S. Riley, S. van Elsland, E. Volz, H. Wang, Y. Wang, X. Xi, C. A. Donnelly, A. C. Ghani, N. M. Ferguson, Estimates of the severity of coronavirus disease 2019: A model-based analysis. *Lancet Infect. Dis.* **20**, 669–677 (2020). doi:10.1016/S1473-3099(20)30243-7 Medline
47. S. M. Moghadas, A. Shoukat, M. C. Fitzpatrick, C. R. Wells, P. Sah, A. Pandey, J. D. Sachs, Z. Wang, L. A. Meyers, B. H. Singer, A. P. Galvani, Projecting hospital utilization during the COVID-19 outbreaks in the United States. *Proc. Natl. Acad. Sci. U.S.A.* **117**, 9122–9126 (2020). doi:10.1073/pnas.2004064117 Medline
48. I. Ngere *et al*., High seroprevalence of SARS-CoV-2 eight months after introduction in Nairobi, Kenya; preprint available at https://europepmc.org/article/ppr/ppr342406 (2021).

ACKNOWLEDGMENTS

We thank staff of the Kenya National Blood Transfusion Service and the blood donors, the county Rapid Response Teams and SARS-CoV-2 PCR testing laboratories, and data management staff of the Ministry of Health, Government of Kenya. This paper is published with permission of the Director of KEMRI.

Funding: This work was supported by the UK Foreign, Commonwealth and Development Office (FCDO) and Wellcome Trust [220985/Z/20/Z]; National Institute for Health Research (NIHR) Global Health Research Project GeMVi [17/63/82] and NIHR Global Health Research Unit on Mucosal Pathogens [16/136/46] JO, using UK Aid from the UK Government to support global health research; Wellcome Trust Intermediate Fellowship awards [201866, 107568] EAO, LIOO; MRC/DFID African Research Leader Fellowship [MR/S005293/1] IMOA, CM; DFID/MRC/NIHR/Wellcome Trust Joint Global Health Trials Award [MR/R006083/1] AA; Wellcome Trust Senior Research Fellowship [214320] and the NIHR Health Protection Research Unit in Immunisation JAGS; Wellcome Trust [220991/Z/20/Z and 203077/Z/16/Z] GMW; UKRI through the JUNIPER modelling consortium [grant number MR/V038613/1]

Author contributions: Brand SPC: Conceptualization, Formal Analysis, Methodology, Software, Writing – Original Draft Preparation, Writing – Review & Editing; Ojal J: Conceptualization, Formal Analysis, Methodology, Software, Writing – Original Draft Preparation, Writing – Review & Editing; Aziza R: Conceptualisation, Formal Analysis, Methodology, Project Administration, Software, Writing – Review & Editing; Were V: Conceptualization, Writing – Review & Editing; Okiro EA: Data Curation, Supervision, Writing – Review & Editing; Kombe IK: Formal Analysis, Methodology, Software, Writing – Review &

Editing; Mburu C: Formal Analysis, Methodology, Software, Writing – Review & Editing; Ogero M: Data Curation, Writing – Review & Editing; Agweyu A: Conceptualization, Project Administration, Funding Acquisition, Writing – Review & Editing; Warimwe GM: Conceptualization, Supervision, Funding Acquisition, Writing – Writing – Review & Editing Nyagwange J, Karanja H, Gitonga JN, Mugo D: Serology methodology, Uyoga S: Data Curation, Investigation, Writing – Review & Editing; Adetifa IMO: Conceptualization, Supervision, Writing – Review & Editing; Scott JAG: Conceptualization, Supervision, Funding Acquisition, Writing – Review & Editing; Otieno E, Otiende M, Murunga N: Data Curation, Writing – Review & Editing; Ochola-Oyier LI: Data Curation, Investigation, Writing – Review & Editing; Agoti CN, Githingi G: Data Curation, Investigation, Writing – Review & Editing; Kasera K: Project Administration, Writing – Review & Editing; Amoth P: Project Administration, Writing – Review & Editing; Mwangangi M: Project Administration, Writing – Review & Editing; Aman R: Project Administration, Writing – Review & Editing; Ng'ang'a W: Project Administration, Writing – Review & Editing; Tsofa B: Project Administration, Writing – Review & Editing; Bejon P: Conceptualization, Writing – Original Draft Preparation, Writing – Review & Editing; Keeling MJ: Conceptualization, Formal Analysis, Funding Acquisition, Methodology, Software, Supervision, Writing – Original Draft Preparation, Writing – Review & Editing; Nokes DJ: Conceptualization, Funding Acquisition, Project Administration, Supervision, Writing – Original Draft Preparation, Writing – Review & Editing Barasa E: Conceptualization, Project Administration, Writing – Original Draft Preparation, Writing – Review & Editing.

Competing interests: The views expressed in this publication are those of the author(s) and not necessarily those of any of the funding agencies. The funders had no role in study design, data collection and analysis, decision to publish, or preparation of the manuscript. All authors declare no competing interests.

Data and materials availability: All code and data for the transmission model underlying this study is accessible at the Github repository (https://github.com/SamuelBrand1/kenya-covid-three-waves and at Zenodo (*31*). All data are available in the main text or the supplementary materials.

This work is licensed under a Creative Commons Attribution 4.0 International (CC BY 4.0) license, which permits unrestricted use, distribution, and reproduction in any medium, provided the original work is properly cited. To view a copy of this license, visit https://creativecommons.org/licenses/by/4.0/. This license does not apply to figures/photos/artwork or other content included in the article that is credited to a third party; obtain authorization from the rights holder before using such material.

SUPPLEMENTARY MATERIALS
science.org/doi/10.1126/science.abk0414
Materials and Methods
Supplementary Text
Figs. S1 to S13
Tables S1 and S2
References (*32*–*48*)
MDAR Reprodubility Checklist
Data S1 to S4

17 June 2021; accepted 4 October 2021
Published online 7 October 2021
10.1126/science.abk0414

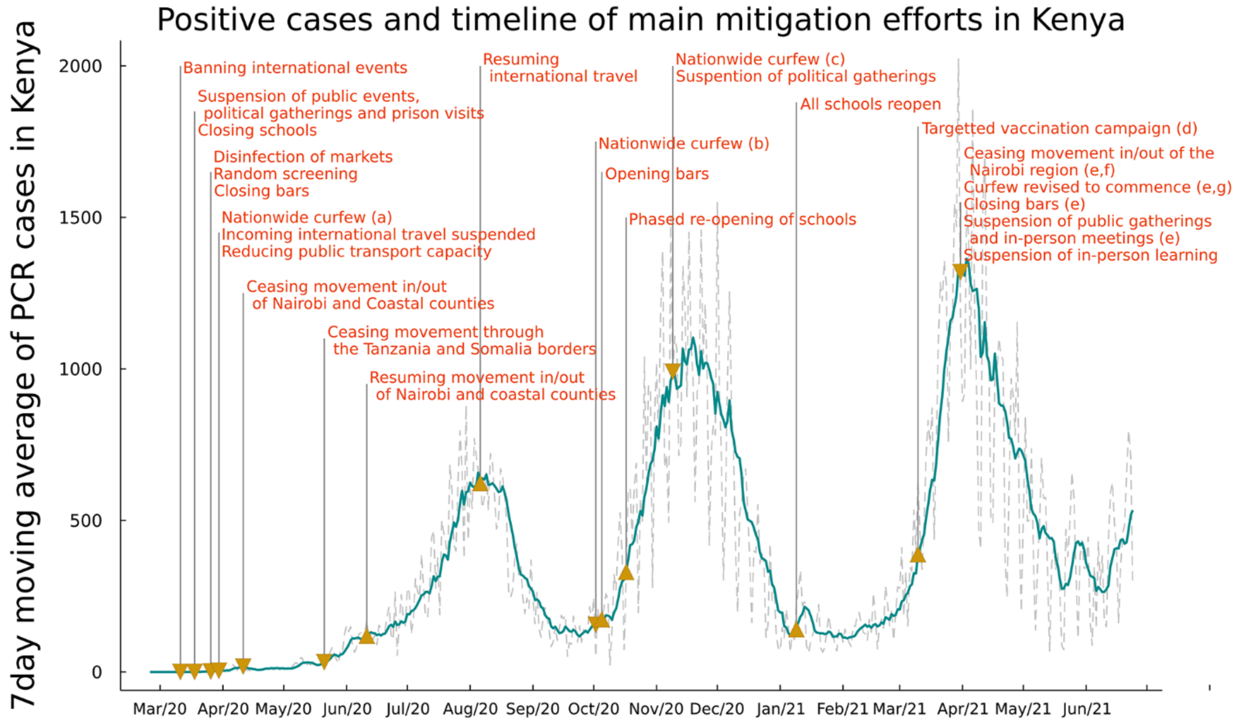

Fig. 1. 7day moving average of daily positive PCR tests from the Kenyan national linelist and a timeline of the main mitigation events applied by the Kenyan government representing tightening (down-arrow) and relaxation (up-arrow) of measures. (a) curfew from 7pm to 5am; (b) curfew from 11pm to 4am; (c) curfew from 10pm to 4am; (d) front line workers and individuals older than 58 years (approximately 1.2m doses); (e) the region includes Nairobi, Kajiado, Machakos, Kiambu, Nakuru; (f) this restricted movement into and out of the block of counties in (e) but not between these counties; (g) curfew from 8pm to 4am.

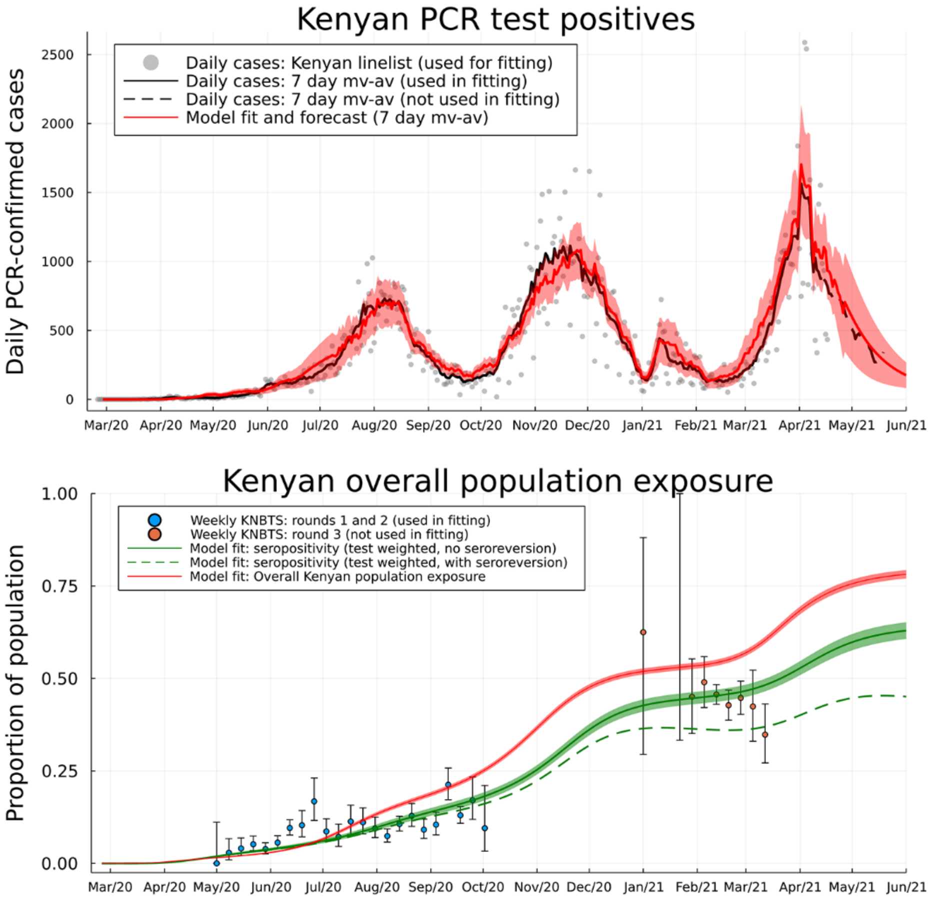

Fig. 2. Daily PCR confirmed COVID-19 cases (top) and weekly serology estimates from KNBTS donors with overall attack rate estimates (bottom). Shown are daily numbers of PCR test positives from the Kenyan national linelist (top; grey dots are daily reports used for fitting the model, curves are 7-day moving averages). The model prediction for the 7-day moving average of daily case incidence (top; red dash curve, shading shows 3-sigma intervals) were derived from inference on the county-specific linelist PCR data and rounds 1 and 2 of the KNBTS serology survey (bottom; blue dots). Predictions before mid-April 2021 are back-calculations using known numbers of PCR tests per day, whereas, after mid-April 2021 model predictions are forecasts which also estimate the number of PCR tests that will occur per day in each county. We show the next month of PCR test positive data, not used in fitting, as a validation of the model short-term predictive accuracy (top; black dashed curve). Back-calculated model estimates of seropositivity (bottom; green solid curve) are shown with round 3 of the KNBTS serology survey data (bottom; red dots, not used in model inference). We also show back-calculated estimates of seropositivity under the assumption that median time to seroreversion (loss of detectable antibodies rather than loss of immunity) from infection was one year. Model estimates of overall Kenyan seropositivity are adjusted from county-specific estimates by weighting by number of serology tests in each county (over KNBTS rounds 1 and 2). The overall estimated Kenyan attack rate (population exposure) is shown as unweighted (bottom; red curve).

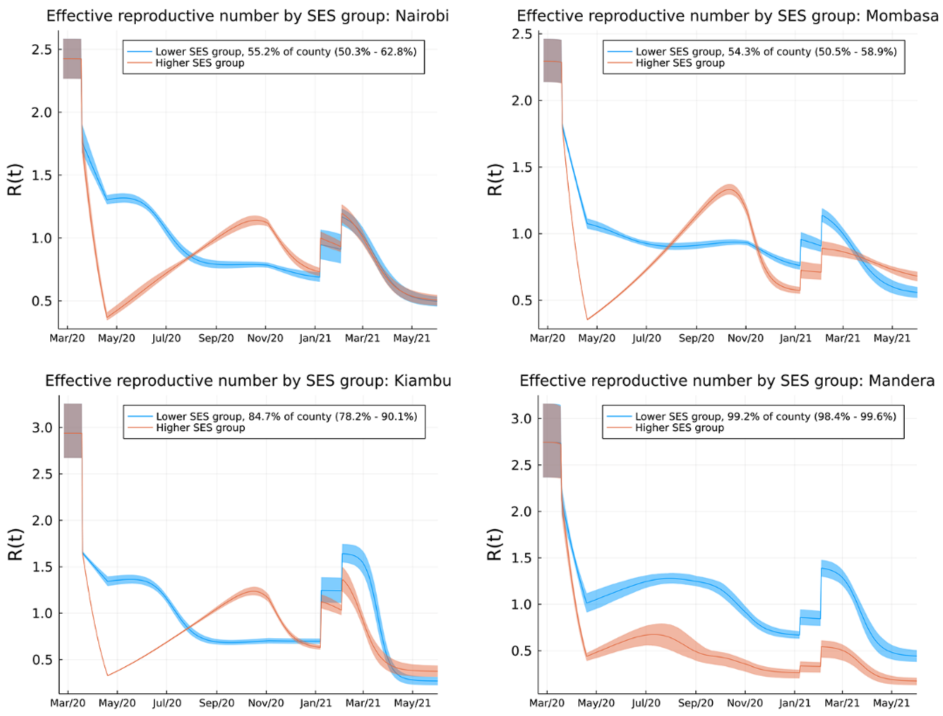

Fig. 3. Effective reproductive number over time (R(t)) for lower and higher SES groups in four representative counties. These include Nairobi (top left), Mombasa (top right), Kiambu (bottom left), and, Mandera (bottom right). Nairobi and Mombasa are Kenya's two largest cities and form fully urban counties, Mandera county has a largely rural population and is remote from the main urban conurbations, Kiambu county borders Nairobi and has a ~60% urban population. The transmission model infers the proportion of the population in each SES group in each county. The highest proportion of higher SES group individuals are inferred to be in Nairobi and Mombasa out of all counties, whereas for Mandera county very close to all individuals are inferred as being in the lower SES group and the model effectively defaults to one group SEIRS transmission. The model inference for R(t) in Kiambu represents a county between these two extremes. In each county, the first discontinuous increase in R(t) is due to schools reopening, and the second is due to more transmissible variants becoming dominant in transmission.

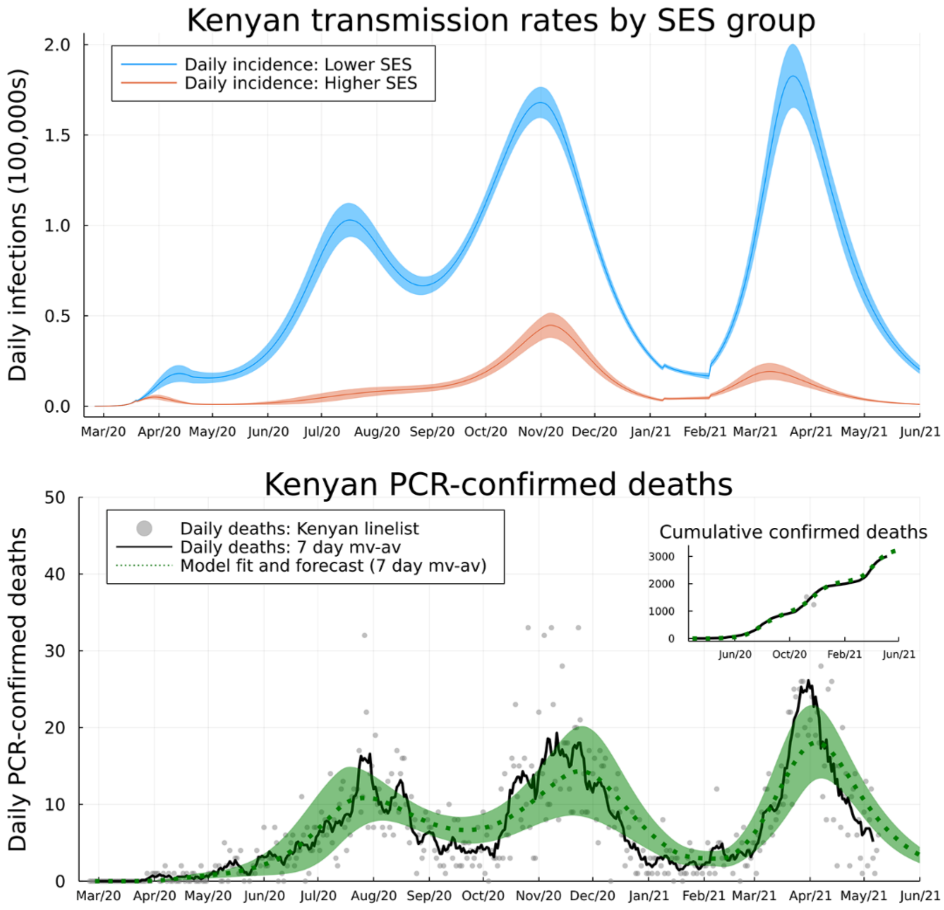

Fig. 4. Model inferred underlying true incidence rates by SES group relative to the whole Kenyan population size (top) and reported PCR-confirmed deaths due to COVID-19 disease (bottom). The size of the upper SES group was estimated to be 11% of the Kenyan population, explaining the lower absolute rate of incidence (red curve) compared to the lower SES group (blue curve). We inferred that the lower SES group have experienced three waves of SARS-CoV-2 transmission, whereas the upper SES group has experienced two. The model fit for seven day moving average (green dashed curve, with shading as 95% PIs) is shown against the seven-day moving average for deaths reported in the Kenyan linelist (black curve). Cumulative observed and fitted deaths are shown in the top-right inset.
