# Supplementary material for: COVID-19 transmission dynamics underlying epidemic waves in Kenya
Source: Science. 2021 Oct 7;374(6570):989–94. doi: 10.1126/science.abk0414 (PMC7612211; doi:10.1126/science.abk0414)
Supplement: Supplementary file 3 — MDAR Reproducibility Checklist [file science.abk0414_mdar_reproducibility_checklist.pdf]

# Materials Design Analysis Reporting (MDAR) Checklist for Authors

The MDAR framework establishes a minimum set of requirements in transparent reporting applicable to studies in the life sciences (see Statement of Task: doi:10.31222/osf.io/9sm4x.). The MDAR checklist is a tool for authors, editors and others seeking to adopt the MDAR framework for transparent reporting in manuscripts and other outputs. Please refer to the MDAR Elaboration Document for additional context for the MDAR framework.

## Materials

| Antibodies | Yes (indicate where provided: page no/section/legend) | n/a |
|---|---|---|
| For commercial reagents, provide supplier name, catalogue number and RRID, if available. | | x |

| Cell materials | Yes (indicate where provided: page no/section/legend) | n/a |
|---|---|---|
| **Cell lines:** Provide species information, strain. Provide accession number in repository **OR** supplier name, catalog number, clone number, **OR** RRID | | x |
| **Primary cultures:** Provide species, strain, sex of origin, genetic modification status. | | x |

| Experimental animals | Yes (indicate where provided: page no/section/legend) | n/a |
|---|---|---|
| **Laboratory animals:** Provide species, strain, sex, age, genetic modification status. Provide accession number in repository **OR** supplier name, catalog number, clone number, **OR** RRID | | x |
| **Animal observed in or captured from the field:** Provide species, sex and age where possible | | x |
| **Model organisms:** Provide Accession number in repository (where relevant) **OR** RRID | | x |

| Plants and microbes | Yes (indicate where provided: page no/section/legend) | n/a |
|---|---|---|
| **Plants:** provide species and strain, unique accession number if available, and source (including location for collected wild specimens) | | x |
| **Microbes:** provide species and strain, unique accession number if available, and source | | x |

| Human research participants | Yes (indicate where provided: page no/section/legend) | n/a |
|---|---|---|
| Identify authority granting ethics approval (IRB or equivalent committee(s), provide reference number for approval. | | x |
| Provide statement confirming informed consent obtained from study participants. | | x |
| Report on age and sex for all study participants. | | x |

## Design

| Study protocol | Yes (indicate where provided: page no/section/legend) | n/a |
|---|---|---|
| For clinical trials, provide the trial registration number **OR** cite DOI in manuscript. | | x |

| Laboratory protocol | Yes (indicate where provided: page no/section/legend) | n/a |
|---|---|---|
| Provide DOI or other citation details if detailed step-by-step protocols are available. | | x |

| Experimental study design (statistics details) | Yes (indicate where provided: page no/section/legend) | n/a |
|---|---|---|
| State whether and how the following have been done**, or** if they were not carried out. | | x |
| Sample size determination | | x |
| Randomisation | | x |
| Blinding | | x |
| Inclusion/exclusion criteria | | x |

| Sample definition and in-laboratory replication | Yes (indicate where provided: page no/section/legend) | n/a |
|---|---|---|
| State number of times the experiment was replicated in laboratory | | x |
| Define whether data describe technical or biological replicates | | x |

| Ethics | Yes (indicate where provided: page no/section/legend) | n/a |
|---|---|---|
| Studies involving human participants: State details of authority granting ethics approval (IRB or equivalent committee(s), provide reference number for approval. | | x |
| Studies involving experimental animals: State details of authority granting ethics approval (IRB or equivalent committee(s), provide reference number for approval. | | x |
| Studies involving specimen and field samples: State if relevant permits obtained, provide details of authority approving study; if none were required, explain why. | | x |

| Dual Use Research of Concern (DURC) | Yes (indicate where provided: page no/section/legend) | n/a |
|---|---|---|
| If study is subject to dual use research of concern, state the authority granting approval and reference number for the regulatory approval | | x |

## Analysis

| Attrition | Yes (indicate where provided: page no/section/legend) | n/a |
|---|---|---|
| State if sample or data point from the analysis is excluded, and whether the criteria for exclusion were determined and specified in advance. | No data points were excluded. | |

| Statistics | Yes (indicate where provided: page no/section/legend) | n/a |
|---|---|---|
| Describe statistical tests used and justify choice of tests. | All statistical inference was performed in the Bayesian paradigm with posterior distribution sampling performed using the "No-U-Turns" algorithm for performing Hamiltonian Markov chain monte carlo (NUTS-HMC). This is now widely considered to be a gold standard approach for parameter inference of mechanistic models of infectious disease transmission.<br><br>The log-likelihood function underlying the Bayesian inference is given on page 11 of the supporting information in section Log-likelihood function. The priors are specific to groups of counties and are given across pages 12-13 in the supporting information text in the section County-specific hierarchy of priors.<br><br>Moreover, we considered multiple alternative mechanistic models. Each of these had posterior distributions for parameters inferred from the same data sources. The model presented in the paper was the model with the most support from the Deviance Information Criterion (DIC). DIC is a commonly used statistical metric used to rank models, after Bayesian inference of posterior distribution, by the data evidence for the model. DIC use for model selection is described in the section Model selection using Deviance Information Criterion on page 16-17 of the supporting information. | |

| Data Availability | Yes (indicate where provided: page no/section/legend) | n/a |
|---|---|---|
| State whether newly created datasets are available, including protocols for access or restriction on access. | For this paper we created an anonymized date and county specific number of positive and negative PCR test dataset. This was generated from the Kenyan Ministry of Health COVID-19 linelist as described in Data Description section on page 2 of the supporting information text. This new dataset is publicly available from the Github repository of code and data for reproducing our findings. | |
| If data are publicly available, provide accession number in repository or DOI or URL. | DOI: 10.5281/zenodo.5541433 | |
| If publicly available data are reused, provide accession number in repository or DOI or URL, where possible. | | x |

| Code Availability | Yes (indicate where provided: page no/section/legend) | n/a |
|---|---|---|
| For all newly generated code and software essential for replicating the main findings of the study: | The modelling and inference methodology code is bundled as a Julia language module **KenyaCoVSD**, along with scripts for generating inference, plotting and analyzing outcomes. | |
| State whether the code or software is available. | This code is available. | |
| If code is publicly available, provide accession number in repository, or DOI or URL. | DOI: 10.5281/zenodo.5541433 | |

## Reporting

| Adherence to community standards | Yes (indicate where provided: page no/section/legend) | n/a |
|---|---|---|
| MDAR framework recommends adoption of discipline-specific guidelines, established and endorsed through community initiatives. Journals have their own policy about requiring specific guidelines and recommendations to complement MDAR. | | |
| State if relevant guidelines (eg., ICMJE, MIBBI, ARRIVE) have been followed, and whether a checklist (eg., CONSORT, PRISMA, ARRIVE) is provided with the manuscript. | | x |
