# Supplementary material for: COVID-19 transmission dynamics underlying epidemic waves in Kenya
Source: Science. 2021 Oct 7;374(6570):989–94. doi: 10.1126/science.abk0414 (PMC7612211; doi:10.1126/science.abk0414)
Supplement: Supplementary file 4 — Data S1 to S4 [file science.abk0414_data_s1_to_s4.zip › science.abk0414_data_s1_to_s4_captions.docx]

Data S1. (Separate file)

**Inferred parameters, SES group-specific fatality-detection estimates, and population exposure by 1^st^ June.** The posterior mean and 95% credible intervals for each model parameter (both transmission and observation models), maximum likelihood estimate for infection fatality-detection ratio, and posterior mean and 95% credible intervals for fraction of population exposed in each county.

Data S2. (Separate file)

**The number of positive, and negative where available, PCR-confirmed swab tests for each county by date of sample collection (21^st^ Feb to 27^th^ May).** A “-17” in the negative swab entry indicates that negative swab tests were not available for that county on that date.

Data S3. (Separate file)

**The number of positive and negative serological results for each county by date of sample collection (21^st^ Feb to 31^st^ March).**

Data S4. (Separate file)

**The number of deaths with a PCR-confirmed swab test for each county by recorded date of death (21^st^ Feb to 27^th^ May).**
